# Digital Biomarkers for Depression Screening With Wearable Devices: Cross-sectional Study With Machine Learning Modeling

**DOI:** 10.2196/24872

**Published:** 2021-10-25

**Authors:** Yuri Rykov, Thuan-Quoc Thach, Iva Bojic, George Christopoulos, Josip Car

**Affiliations:** 1 Neuroglee Therapeutics Singapore Singapore; 2 Department of Psychiatry The University of Hong Kong Hong Kong SAR China (Hong Kong); 3 Centre for Population Health Sciences Lee Kong Chian School of Medicine Nanyang Technological University Singapore Singapore; 4 Division of Leadership, Management and Organisation Nanyang Business School, College of Business Nanyang Technological University Singapore Singapore; 5 Department of Primary Care and Public Health School of Public Health Imperial College London London United Kingdom

**Keywords:** depression, digital biomarkers, screening, wearable electronic device, fitness tracker, circadian rhythm, rest-activity rhythm, heart rate, machine learning

## Abstract

**Background:**

Depression is a prevalent mental disorder that is undiagnosed and untreated in half of all cases. Wearable activity trackers collect fine-grained sensor data characterizing the behavior and physiology of users (ie, digital biomarkers), which could be used for timely, unobtrusive, and scalable depression screening.

**Objective:**

The aim of this study was to examine the predictive ability of digital biomarkers, based on sensor data from consumer-grade wearables, to detect risk of depression in a working population.

**Methods:**

This was a cross-sectional study of 290 healthy working adults. Participants wore Fitbit Charge 2 devices for 14 consecutive days and completed a health survey, including screening for depressive symptoms using the 9-item Patient Health Questionnaire (PHQ-9), at baseline and 2 weeks later. We extracted a range of known and novel digital biomarkers characterizing physical activity, sleep patterns, and circadian rhythms from wearables using steps, heart rate, energy expenditure, and sleep data. Associations between severity of depressive symptoms and digital biomarkers were examined with Spearman correlation and multiple regression analyses adjusted for potential confounders, including sociodemographic characteristics, alcohol consumption, smoking, self-rated health, subjective sleep characteristics, and loneliness. Supervised machine learning with statistically selected digital biomarkers was used to predict risk of depression (ie, symptom severity and screening status). We used varying cutoff scores from an acceptable PHQ-9 score range to define the depression group and different subsamples for classification, while the set of statistically selected digital biomarkers remained the same. For the performance evaluation, we used k-fold cross-validation and obtained accuracy measures from the holdout folds.

**Results:**

A total of 267 participants were included in the analysis. The mean age of the participants was 33 (SD 8.6, range 21-64) years. Out of 267 participants, there was a mild female bias displayed (n=170, 63.7%). The majority of the participants were Chinese (n=211, 79.0%), single (n=163, 61.0%), and had a university degree (n=238, 89.1%). We found that a greater severity of depressive symptoms was robustly associated with greater variation of nighttime heart rate between 2 AM and 4 AM and between 4 AM and 6 AM; it was also associated with lower regularity of weekday circadian rhythms based on steps and estimated with nonparametric measures of interdaily stability and autocorrelation as well as fewer steps-based daily peaks. Despite several reliable associations, our evidence showed limited ability of digital biomarkers to detect depression in the whole sample of working adults. However, in balanced and contrasted subsamples comprised of depressed and healthy participants with no risk of depression (ie, no or minimal depressive symptoms), the model achieved an accuracy of 80%, a sensitivity of 82%, and a specificity of 78% in detecting subjects at high risk of depression.

**Conclusions:**

Digital biomarkers that have been discovered and are based on behavioral and physiological data from consumer wearables could detect increased risk of depression and have the potential to assist in depression screening, yet current evidence shows limited predictive ability. Machine learning models combining these digital biomarkers could discriminate between individuals with a high risk of depression and individuals with no risk.

## Introduction

### Background

Depression is the third-largest contributor to years lost to disability and affects 264 million people globally [1]. Despite its high prevalence, depression remains undiagnosed and untreated in half of all cases [2,3]. At the same time, the evolving COVID-19 pandemic and related economic crisis are worsening the population’s mental health [4-6].

Wearable activity trackers are increasingly widely used and provide an opportunity to harness sensor data for detection of different health conditions [7]. Wearable trackers can monitor physiological and behavioral data, including steps, heart rate, energy expenditure, sleep patterns, respiration rate, blood oxygen saturation, skin temperature, and skin conductance, among others. Close interrelationships between everyday behavior, physiology, and mental well-being makes digital phenotyping with wearables especially attractive for detection of mental disorders and discovering respective digital biomarkers [8-11]. These digital biomarkers could be used for risk prediction of depression and to scale up population mental health screening. Moreover, due to the unprecedented granularity of available data, digital phenotyping can advance our understanding of etiology and subtypes of mental disorders and complement established diagnostic criteria. Complementary smartphone apps could be further used for prevention and treatment of mental disorders, to deliver digital health interventions and personalized cognitive behavioral therapy [12-14]. In this work, we focus on digital biomarkers based on wearable sensor data for depression screening.

### Related Work

Current evidence is mainly comprised of studies investigating separate associations between depressive symptomatology and various actigraphy and sensor-based metrics, which capture meaningful aspects of behavior and physiology, to reveal objective risk or diagnostic markers. These aspects include physical activity, sleep characteristics, circadian rhythms, and physiological parameters. Systematic reviews and meta-analyses of actigraphy studies demonstrated that patients with depression were significantly less physically active than healthy controls [15,16]. Meta-analyses of prospective cohort studies showed that physical activity had a protective effect against the future onset of depression [17], while sedentary time increased the risk of depression [18]. For example, odds of depression development were 1.8 to 2.7 times lower in participants with more time spent in moderate to vigorous physical activity compared to the least active participants [19].

Sleep disturbance is another common symptom and risk factor for depression that can be measured objectively together with other sleep characteristics. Meta-analyses of polysomnography (PSG) studies showed that depression is associated with less total sleep time (TST), increased sleep onset latency (SOL), lower sleep efficiency (SE), lower duration and fraction of slow-wave sleep, lower fraction of light sleep, reduced latency between sleep onset and onset of rapid eye movement (REM) sleep, greater duration and fraction of REM sleep, greater number of awakenings, and increased wake after sleep onset (WASO) time compared to healthy controls [20,21]. Although PSG is a gold standard for sleep measurement, contemporary wearables can measure sleep and identify sleep stages. A systematic review of actigraphy studies suggested that the most persistent sleep abnormality in depressive patients is longer WASO, whereas other parameters had mixed effects [16]. Separate actigraphy studies showed lower SE, longer SOL, later sleep offset [22,23], and a greater sleep fragmentation in adults with depression [24].

Contrary to measuring physical activity and sleep as separate factors, circadian rhythms characterize the activity pattern of a full 24-hour cycle. Circadian rhythm metrics quantify regularity, shape, and timing of repeated daily processes and can be derived from fine-grained wearable data. Actigraphy studies suggested that people with clinically significant depressive symptoms had lower mesor, a rhythm-adjusted mean activity level [24-26]; reduced total locomotor activity [27,28]; lower amplitude [25,26]; shorter active periods [29,30]; delayed acrophase [22,30]; and less robust circadian activity rhythms [22,25,31]. Longitudinal studies showed that lower rhythm robustness predicted worsening of depressive symptoms in the future [26]. Depressive symptoms were also associated with heart rate–based mesor [32] and amplitude [32] as well as increased heart rate in the night and morning hours [33]. Few studies using nonparametric rhythm measures demonstrated that participants with more severe depressive symptoms had lower interdaily stability (IS) and higher intradaily variability (IV) of circadian activity rhythm [31,34], reduced relative amplitude (RA) between the highest and the lowest activity levels [28,35], and reduced RA of skin temperature [36]. Although findings on separate circadian rhythm indicators are mixed, the evidence suggests that depression is often associated with disturbed, irregular, and delayed circadian behavioral and physiological rhythms.

Another stream of evidence is comprised of a few studies that developed predictive models for depression detection from multimodal wearable data using data-driven approaches and machine learning. For example, Jacobson et al extracted 9929 digital markers using signal processing methods from a publicly available actigraphy data set of 55 individuals to detect people with depressive disorder [37]. A machine learning model correctly predicted the diagnostic status in 89% of the cases with a sensitivity of 94% and a specificity of 83% using leave-one-cohort-out cross-validation. Only spectral density features were important predictors [38]. In another study, authors extracted distribution characteristics of wearable data, including acceleration, skin conductance, and temperature, collected for 1 month and sampled from different time windows within 24-hour cycles, which resulted in 204 features in total [39]. A machine learning model with this feature set reached 87% accuracy in classifying 47 participants with high or low mental health scores using leave-one-out cross-validation. Nighttime skin conductance features were most important in prediction models. Tazawa et al extracted distribution characteristics of per-hour data from multidimensional wearable data, including steps, energy expenditure, body motion, heart rate, skin temperature, sleep time, and ultraviolet light, which resulted in 63 features [33]. A machine learning model with features based on a 7-day period achieved an accuracy of 76%, a sensitivity of 73%, and a specificity of 79% in prediction of depression screening status in 86 participants using 10-fold cross-validation. Authors found that features of skin temperature and sleep were most important for the model’s predictive ability.

### Objectives

The first stream of evidence from actigraphy and PSG studies lacks research on the predictive ability of various metrics to assess the risk of depression, whereas the other stream of data-driven studies often ignores established risk markers and leaves associations between depressive symptomatology and wearable data uninterpreted. Absence of robust and interpretable digital biomarkers complicates further development of a comprehensive and explainable algorithm for depression screening in the general population. To address these gaps, we examined the associations between depressive symptomatology and digital biomarkers based on sensor data from consumer-grade fitness trackers, including established and novel markers, and explored the predictive ability of these digital biomarkers in depression screening.

## Methods

### Study Design and Participants

In a cross-sectional study, we collected data from a multiethnic working population in Singapore. A total of 290 adult volunteers (aged ≥21 years) among full-time employees of Nanyang Technological University (NTU) were recruited to participate in the study from August to October 2019. Participants responded to online questionnaires and wore activity trackers for at least 14 days. Subjects received financial compensation for participating in the study. The study protocol and informed consent form were approved by the NTU Institutional Review Board (application reference: RB-2016-03-033).

Fitbit Charge 2 devices—consumer-grade fitness trackers—were used for data collection. The accuracy of Fitbit data has been investigated in several studies [40-44]. According to a systematic review [42], studies have consistently indicated that Fitbit devices were likely to provide accurate measures of daily step counts. However, energy expenditure is less accurately estimated by Fitbit devices. In general, Fitbit wearables have similar accuracy for activity assessment as research-grade devices, but they overestimate moderate to vigorous physical activity in free-living conditions. According to a recent meta-analysis, sleep-staging Fitbit devices, such as the Charge 2, showed no significant difference in measured values of WASO, TST, and SE, but they slightly underestimated SOL in comparison to the gold standard PSG [45]. Also sleep-stage transition dynamics measured by Fitbit devices were found to be inaccurate compared to PSG [46]. Participants were instructed to wear trackers all the time and to remove them only when taking a shower or charging. Data from trackers were synchronized with the Fitbit mobile app and further transferred to the Fitabase server.

### Depression Screening and Self-reported Covariates

We used validated self-report questionnaires for depression screening and to collect sociodemographic, lifestyle, and health characteristics. REDCap (Research Electronic Data Capture), the research-grade online survey platform, was used for survey administration. The 9-item Patient Health Questionnaire (PHQ-9) was used for depression screening. Participants completed the PHQ-9 at the beginning and at the end of the observation period. We used different cutoff points and approaches to define provisionally depressed participants in the analysis based on common standards and the actual distribution of scores across two assessments. The average PHQ-9 score of two assessments was used in statistical analysis.

We collected a range of covariates to control for possible confounders. Demographics, including age, gender, ethnic group (ie, Chinese, Malay, Indian, or other), marital status, education (ie, university degree or below), and income level (ie, above or below SGD 4000 [US $3000]), were collected. Additionally, we collected data on alcohol consumption, smoking status, and self-rated health. We assessed sleep characteristics, including subjective sleep quality, using the Pittsburgh Sleep Quality Index [47]; sleep hygiene using the Sleep Hygiene Index [48]; daytime sleepiness using the Epworth Sleepiness Scale [49]; and perceived loneliness using the revised UCLA (University of California, Los Angeles) Loneliness Scale [50]. All covariates were collected at the beginning of activity tracking.

### Wearable Data Preprocessing

Fitbit devices measure steps; energy expenditure, measured in metabolic equivalents (METs); and heart rate, measured in beats per minute (bpm), and they identify sleep stages (ie, wake, light, deep, or REM sleep). Steps and energy expenditure are available at by-the-minute intervals, sleep stages are available at 30-second intervals, and heart rate data are available at 5- or 10-second intervals. We examined the completeness of the data set using heart rate data to verify the actual device use time as the most sensitive to inappropriate use or nonuse. Missing and complete time points of heart rate data were determined from the full period of participant tracking within the study. Time points of activity and sleep data corresponding to complete heart rate data points were sampled and considered as clean data. We used data from days with at least 20 hours of complete recording for further analysis. Participants with a minimum of 10 complete days were included for further analysis (see Multimedia Appendix 1 for the distribution of complete days among participants). Outliers were identified using the Tukey rule for outliers—outliers are values more than 1.5 times the IQR from the quartiles—either below the first quartile or above the third quartile.

### Extraction of Digital Biomarkers

We extracted a range of digital biomarkers characterizing physical activity, sleep, circadian rhythms, and physiological parameters from the raw data. This set of digital biomarkers relied on previous findings and was substantially extended with novel metrics hypothetically indicative of depressive symptoms [15-18,20,21,24-26,30,32,34,36].

First, we extracted physical activity metrics based on steps and energy expenditure data, including daily steps, time spent at different intensity levels of physical activity, and sedentary time. The daily durations of light, moderate, and vigorous physical activity were determined according to the physical activity guidelines of the US Centers for Disease Control and Prevention [51], where moderate activity corresponds to energy expenditures from 3.0 to 6.0 METs, vigorous activity is above 6.0 METs, and light physical activity is below 3.0 METs. We sampled minutes within these intervals separately and calculated the mean daily sum of these minutes. Sedentary time was defined as any waking behavior with energy expenditure less than 1.5 METs [52]. Hence, to determine sedentary time, we excluded all sleep intervals and calculated a daily mean of total minutes with ≤1.5 METs. Daily steps and sedentary time were calculated for all days, and for weekdays and for weekends separately.

Second, we extracted sleep metrics, including average values and coefficients of variation (CVs) of time in bed, TST, SE, SOL, and WASO. TST is the difference between the length of time in bed and the length of wake time, and SE is the ratio of TST to time in bed. SOL is the length of time in minutes until the first minute of sleep onset from bedtime (ie, from the beginning of a sleep episode). WASO was calculated as the number of wake minutes in the middle half of a sleep episode (ie, in the second and third quartiles of a sleep episode). Additionally, we computed the mean and SD of sleep offset and sleep midpoint time as measured in hours since midnight. All sleep metrics were calculated for all days, and for weekdays separately.

Third, we extracted cosinor-based and nonparametric measures of circadian rhythms using steps and heart rate data. These metrics were extracted based on data from all days and based on weekdays only separately. Cosinor-based metrics were estimated using the extended cosinor model with antilogistic transformation and included mesor, acrophase, amplitude, pseudo-*F* statistic, and α and β parameters. Nonparametric measures included IS, IV, interdaily coefficient of variation (ICV), diurnal activity level (mean activity of the 10 consecutive most active hours of the day [M10]), nocturnal activity level (mean value of the 5 consecutive least active hours of the day [L5]), and RA. In addition, we calculated lagged autocorrelation and peak detection–based metrics. Mesor is a rhythm-adjusted mean value that estimates central tendency of the distribution of an oscillating variable, with lower values indicating reduced activity levels [53]. Acrophase is the time of day of the cosine curve peak. Amplitude is the difference between the peak value of the curve and mesor, where lower amplitude indicates a more dampened rhythm. The pseudo-*F* statistic is the goodness-of-fit estimate of the fitted model, which indicates so-called robustness of the rhythm. α is the relative width of the curve at the middle of the peak, and β is an indicator of the steepness of the rise and fall of the curve.

IS is a measure of stability and regularity of activity patterns across a series of 24-hour cycles and is calculated as the ratio of variance of the average 24-hour activity profile to the total variance of data aggregated by the hour from all days [54]. Higher IS indicates more stable, more regular circadian rhythm:



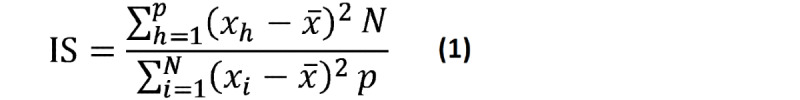



where *N* is the total number of data points, *p* is the number of data points per day (24 in this case), *x_h_* represents values of each hour from the mean 24-hour profile, *x_i_* represents each given hour of raw data, and 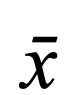 is the mean of all data.

IV quantifies the fragmentation of rest and activity periods within a 24-hour cycle and is calculated as the mean square of differences between successive hourly aggregated data normalized by the total variance of all days [54]. Higher IV indicates a more fragmented rhythm and reflects shorter alternating periods of rest and activity rather than one extended active period and one extended rest period:



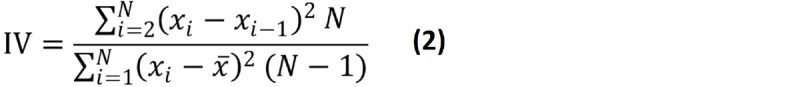



where *N* is the total number of data points, *x_i_* represents the value of a given hour, and 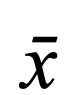 is the mean of all data.

RA reflects the difference between the most and the least active periods and is calculated as the difference between M10 and L5 divided by sum of M10 and L5, where higher values show greater amplitude:



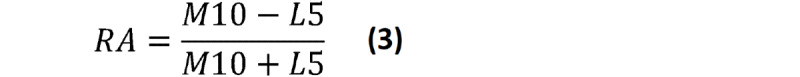



ICV is a novel rhythm stability measure and is calculated as the 24-hour mean of CVs, which is the ratio of the SD to the mean in each hour between days. A higher coefficient indicates higher variation and less stable rhythm. We proposed this metric as an alternative to IS, which aims to assess the same phenomena with a different approach:



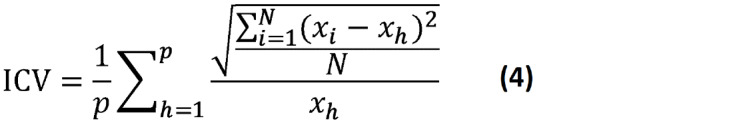



where *p* is the number of data points per day (24 in this case; data are aggregated by the hour), *x_i_* represents values corresponding to each hour from all days, *x_h_* represents values of each hour from the mean 24-hour profile, and *N* is the number of days.

Autocorrelation is another measure of rhythm stability, calculated as the lagged autocorrelation of time series. Autocorrelation was calculated for time series aggregated into 15-minute, 30-minute, and 60-minute intervals with a day-length lag:



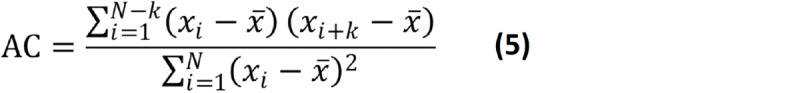



where AC is autocorrelation, *k* is the day-length lag (eg, 24 if data are aggregated by the hour), *x_i_* represents values of each interval, 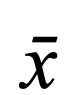 is the mean of all data, and *N* is total number of data points.

We applied the robust peak-detection algorithm based on *z* scores to times series to identify peaks in steps and heart rate data [55]. We calculated the daily mean number of peaks and SD.

Finally, extracted heart rate–based metrics included the overall average heart rate, resting heart rate (RHR), delta heart rate, daytime and nighttime heart rate, a variation of these measures using SD and CV, and the root mean square of successive differences (RMSSD) of heart rate. RHR was calculated as the average heart rate for 15-minute intervals with zero steps. Daytime heart rate was obtained by averaging heart rate values between 2 PM and 4 PM, whereas nighttime heart rate was obtained by averaging values sampled from three consecutive 2-hour intervals: 12 AM to 2 AM, 2 AM to 4 AM, and 4 AM to 6 AM. Unlike daytime heart rate, nighttime heart rates were sampled from more time intervals because nocturnal physiological processes seemed to be more sensitive to depression [56]. We calculated SDs and CVs for all heart rates. Delta heart rate is the difference between average heart rate and RHR. RMSSD values of heart rate were calculated from raw data and from data aggregated into hourly intervals.

In total, we extracted 126 digital biomarkers. The full list of digital biomarkers with detailed descriptions is presented in Multimedia Appendix 2.

### Statistical Analysis and Predictive Modeling With Machine Learning

Spearman rank correlation was used to evaluate the strength of associations between digital biomarkers and severity of depressive symptoms. The false discovery rate (FDR) was used for multiple testing correction of *P* values [57]. A multiple hierarchical linear regression was used to determine the strength of association between digital biomarkers and severity of depressive symptoms adjusted for covariates, including sociodemographics, alcohol consumption, smoking, self-rated health, subjective sleep characteristics, and loneliness.

Next, we trained a series of supervised machine learning models predicting symptom severity and depression screening status of participants (ie, depressed or healthy) to evaluate the predictive ability of digital biomarkers identified at the previous step of statistical analysis. Digital biomarkers were selected based on Spearman correlation at different levels of significance, including *P*<.01, *P*<.05, and *P* values between .01 and .05. Extreme gradient boosting algorithm (DART [Dropouts meet Multiple Additive Regression Trees]) was used for model training, as it was the most efficient method in similar studies [33,37,58]. For performance evaluation of the symptom severity prediction model, we used repeated k-fold cross-validation with 4 folds and 25 repeats and obtained *R*^2^, mean absolute error (MAE), and root mean square error (RMSE) for holdout folds. For evaluation of the model predicting depression screening status, we used k-fold cross-validation with 4 folds and 25 repeats and obtained accuracy, sensitivity, specificity, positive predictive value, negative predictive value, Cohen κ, and area under the curve (AUC). The k-fold cross-validation was used as an alternative to performance evaluation with the testing set, which is usually between 20% and 30% of a sample and, therefore, using 4-fold cross-validation corresponds to 25% of a sample in holdout folds for performance evaluation. Relative feature importance, which indicates the contribution of each predictor in the overall improvement of classification accuracy across all decision trees, was reported. We used RStudio from R (version 3.6.0; The R Foundation) for all analyses. R scripts for feature extraction and statistical analyses are available in Multimedia Appendix 3.

## Results

### Characteristics of the Data and Participants

We collected 5180 days of observational data in total, or 17 days and 20 hours per participant, on average. The mean compliance with wearing the activity tracker among participants within the observation period was 84.8% (SD 10.4), whereas the total fraction of complete data was 84.0% (6,265,469 minutes of complete data from 5180 days). A total of 77.7% (4025/5180) of days contained at least 20 hours of complete data; however, only 277 participants out of 290 (95.5%) had at least 10 days with enough data (see Table S1 in Multimedia Appendix 1). Thus, 5,464,248 minutes of complete data from 3948 days in total (83.7%) were included for further analysis, and 801,221 minutes of complete data (12.8%) were discarded. Further, we identified and excluded outliers: 1 participant was excluded due to outlying heart rate, 4 participants were excluded due to exceeding average steps, and another 5 participants were excluded due to insufficient sleep data with less than three nights on weekdays. The final sample comprised of 267 participants with 14 days of tracking data, on average; the participant flowchart is presented in Multimedia Appendix 4.

**Figure 1:**
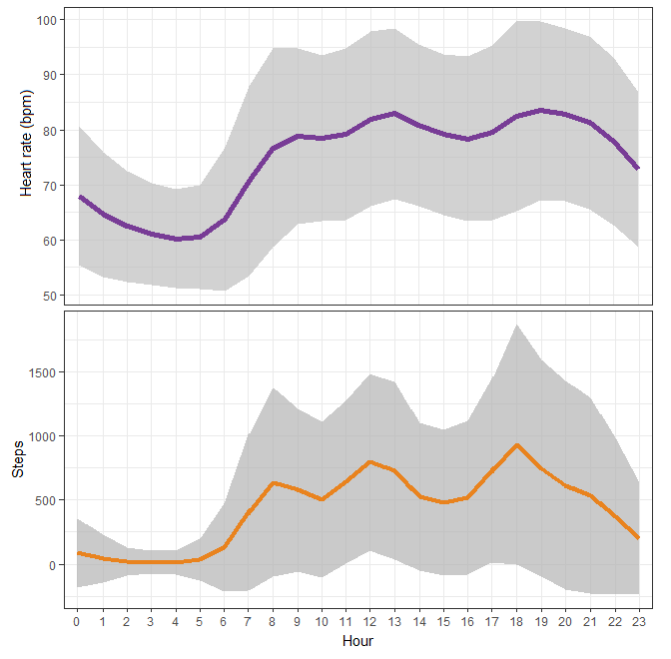
Average 24-hour profiles of heart rate and steps measured by the wearable activity tracker. The purple and orange lines represent the means and the gray shaded areas represent the SDs. bpm: beats per minute.

The mean age of the participants was 33 (SD 8.6, range 21-64) years. Out of 267 participants, a mild female bias was displayed (n=170, 63.7%). The majority of the participants were Chinese (n=211, 79.0%), single (n=163, 61.0%), with a university degree (n=238, 89.1%), and with a monthly income below SGD 4000 (US $3000; n=154, 57.7%). Only 15 participants (5.6%) were current smokers, while 158 participants (59.2%) reported drinking alcohol. Finally, most participants rated their health as “good” (n=124, 46.4%) or “very good” (n=105, 39.3%); fewer participants rated their health as “fair” (n=21, 7.9%) and the lowest number of participants felt that their health was “excellent” (n=17, 6.4%). The mean step count was 10,252 (SD 2822) steps per day and the mean heart rate was 74 (SD 7.1) bpm. Figure 1 shows the smoothed 24-hour daily profile of heart rate and steps averaged in 1-hour windows in the study sample. Table 1 summarizes the characteristics of the participants.

**Table 1:** Sociodemographic characteristics, self-reported health outcomes, and basic wearable metrics of participants.

Participant characteristics		Value (N=267)	
Age (years), mean (SD)		32.8 (8.6)	
**Gender, n (%)**			
	Male	97 (36.3)	
	Female	170 (63.7)	
**Ethnicity, n (%)**			
	Chinese	211 (79.0)	
	Indian	22 (8.2)	
	Malay	10 (3.7)	
	Other	24 (9.0)	
**Marital status, n (%)**			
	Married	104 (39.0)	
	Single	163 (61.0)	
**Education, n (%)**			
	University degree	238 (89.1)	
	Below university degree	29 (10.9)	
**Monthly income (SGD^a^), n (%)**			
	<4000	154 (57.7)	
	≥4000	113 (42.3)	
**Alcohol consumption, n (%)**			
	No	109 (40.8)	
	Yes	158 (59.2)	
**Smoking, n (%)**			
	Current smoker	15 (5.6)	
	Nonsmoker	252 (94.4)	
**Overall self-rated health, n (%)**			
	Fair	21 (7.9)	
	Good	105 (46.4)	
	Very good	124 (39.3)	
	Excellent	17 (6.4)	
Sleep Hygiene Index score, mean (SD)		16.9 (5.7)	
Pittsburgh Sleep Quality Index score, mean (SD)		5.4 (2.7)	
Daytime sleepiness score (ESS^b^), mean (SD)		7.2 (4.2)	
Loneliness score (UCLA^c^ Loneliness Scale), mean (SD)		37.8 (9.8)	
Complete actigraphy days, mean (SD)		14.2 (2.1)	
Steps per day, mean (SD)		10,252 (2822)	
Heart rate (beats per minute), mean (SD)		74.4 (7.1)	

^a^A currency exchange rate of SGD 1=US $0.75 is applicable.

^b^Epworth Sleepiness Scale.

^c^UCLA: University of California, Los Angeles.

**Figure 2:**
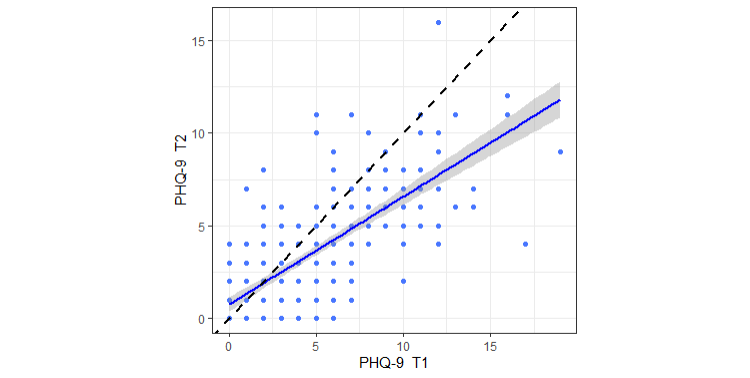
Scatterplot of 9-item Patient Health Questionnaire (PHQ-9) scores at two assessments (T1 and T2). The blue line is the linear projection of the relationship between two scores with CIs and the dashed diagonal line represents no change in scores between two assessments.

Table 2 and Figure 2 display the distribution of PHQ-9 scores across two assessments and show the change in scores over a 2-week period. PHQ-9 scores are highly and linearly correlated (*r*=0.73, *P*<.001), showing that the change in depressive symptoms over time is quite smooth and gradual rather than quick and sharp. However, participants experienced an overall decrease in depressive symptoms over time; for example, 20 participants with moderate (score 10-14) depressive symptoms at the first assessment shifted to the mild range (score 5-9) at the second assessment, while just 5 participants with mild symptoms at the first assessment appeared to have moderate symptoms at the second assessment. Figure 2 shows the same trend. Hence, using the widely adopted cutoff score of ≥10 for both assessments significantly reduced the prevalence rate of depressed participants: only 8 participants had PHQ-9 scores of ≥10 at both assessments. Given that the overall change in symptom severity is gradual, we used less strict criteria and defined a participant as provisionally depressed if he or she scored 10 at one or both assessments and was otherwise healthy. Having a PHQ-9 score of ≥10 at any time point means that the score at an adjacent time point is likely to be comparable in magnitude. In total, 38 out of 267 (14.2%) participants had PHQ-9 scores of ≥10 at either of the two assessments and were identified as depressed. In addition, we used alternative cutoff points to define depression in participants. First, a meta-analysis by Manea et al [59] showed that a PHQ-9 cutoff score of ≥8 also has acceptable screening properties; in our sample, there were 22 (8.2%) participants who had scores of ≥8 at both assessments. Second, given the linearity of PHQ-9 change over time, we used an average PHQ-9 score of ≥8 as another criterion; 42 (15.7%) participants had an average PHQ-9 score ≥8.

**Table 2:** The distribution of PHQ-9 scores across two assessments.

PHQ-9^a^ score at the first assessment	Participants within each category of PHQ-9 scores at the second assessment (N=267), n (%)				
	Normal: score of 0-4	Mild: score of 5-9	Moderate: score of 10-14	Moderately severe: score of ≥15	Total
Normal: score of 0-4	143 (53.6)	14 (5.2)	0 (0)	0 (0)	157 (58.8)
Mild: score of 5-9	32 (12.0)	40 (15.0)	5 (1.9)	0 (0)	77 (28.8)
Moderate: score of 10-14	3 (1.1)	20 (7.5)	5 (1.9)	1 (0.4)	29 (10.9)
Moderately severe: score of ≥15	1 (0.4)	1 (0.4)	2 (0.7)	0 (0)	4 (1.5)
Total	179 (67.0)	75 (28.0)	12 (4.5)	1 (0.4)	267 (100)

^a^PHQ-9: 9-item Patient Health Questionnaire.

### Associations Between Digital Biomarkers and Depressive Symptoms

Correlation analysis revealed that 36 digital biomarkers were significantly associated with the average PHQ-9 score—absolute coefficients of Spearman rank correlation were weak and varied from 0.12 to 0.26—and only 17 of them remained significant predictors after the FDR correction (Table 3). Severity of depressive symptoms was correlated to variation in nighttime heart rate in several time intervals, regularity of circadian rhythms measured with nonparametric and cosinor measures, daily peaks, and timing and variation in sleep offset and midpoint. Physical activity metrics were not associated with PHQ-9 scores.

**Table 3:** Correlation between digital biomarkers and 9-item Patient Health Questionnaire scores.

Category and digital biomarkers		Spearman rank correlation coefficient	*P* value	Adjusted *P* value^a^
**Heart rate metrics**				
	DHR^b^.cv^c^	0.121	.047	.18
	NHR^d^.0204.sd^e^	0.262	<.001	.001
	NHR.0204.cv	0.257	<.001	.001
	NHR.0406.sd	0.182	.003	.04
	NHR.0406.cv	0.185	.002	.04
	NHR.0002.sd	0.149	.01	.08
	NHR.0002.cv	0.138	.02	.11
**Circadian rhythm metrics: nonparametric**				
	IS^f^.st^g^.wd^h^	–0.165	.007	.049
	IS.hr^i^.wd	–0.199	.001	.02
	AC^j^.st.60m^k^	–0.125	.04	.15
	AC.st.15m	–0.175	.004	.04
	AC.st.30m	–0.155	.01	.07
	AC.st.60m.wd	–0.159	.009	.06
	AC.st.15m.wd	–0.177	.004	.04
	AC.st.30m.wd	–0.177	.004	.04
	AC.hr.60m.wd	–0.150	.01	.08
	AC.hr.30m.wd	–0.144	.02	.08
	ICV^l^.st.wd	0.176	.004	.04
	ICV.hr	0.177	.004	.04
	ICV.hr.wd	0.237	<.001	.004
	peaks.st	–0.205	<.001	.02
	peaks.st.wd	–0.202	<.001	.02
**Circadian rhythm metrics: cosinor based**				
	acro^m^.st	0.133	.03	.12
	F.st.wd	–0.121	.046	.17
	beta.hr	0.169	.006	.04
	acro.hr	0.146	.02	.09
	F.hr	–0.126	.04	.15
	beta.hr.wd	0.126	.04	.15
	acro.hr.wd	0.154	.01	.07
	F.hr.wd	–0.137	.03	.11
**Sleep metrics**				
	sleep.offset	0.144	.02	.09
	sleep.midpoint	0.172	.005	.04
	sleep.offset.wd	0.134	.03	.12
	sleep.offset.wd.sd	0.199	.001	.02
	sleep.midpoint.wd	0.146	.02	.09
	sleep.midpoint.wd.sd	0.146	.02	.09

^a^*P* values were adjusted for multiple testing correction. Only significant associations were reported.

^b^DHR: daytime heart rate between 2 PM and 4 PM.

^c^cv: coefficient of variation.

^d^NHR: nighttime heart rate in a specified 2-hour time interval; 2 AM-4 AM (0204), 4 AM-6 AM (0406), and 12 AM-2 AM (0002).

^e^sd: standard deviation.

^f^IS: interdaily stability.

^g^st: steps based.

^h^wd: weekdays based.

^i^hr: heart rate based.

^j^AC: autocorrelation.

^k^m: minutes; 15-minute, 30-minute, or 60-minute time interval in which raw data were aggregated.

^l^ICV: interdaily coefficient of variation.

^m^acro: acrophase.

Further linear regression analysis showed that 11 digital biomarkers—seven nonparametric measures of weekday circadian rhythm regularity, based on steps and heart rate, and four metrics of heart rate variation at nighttime intervals—were significantly associated with severity of depressive symptoms independent of sociodemographic confounders, including age, gender, ethnicity, marital status, education, and income levels (see Table S2 in Multimedia Appendix 1). Only three digital biomarkers—weekday steps–based IS, autocorrelation (based on 15-minute intervals), and CV of heart rate between 4 AM and 6 AM—were consistently associated with symptom severity independent of all confounders, which additionally included alcohol consumption, smoking, self-rated health, loneliness, and subjective sleep characteristics (Table 4). Weekday steps–based IS and autocorrelation were negatively associated with PHQ-9 scores (for IS in the fully adjusted model: β=−0.30 per 10% change, 95% CI −0.51 to −0.08, *P*=.01; for autocorrelation: β=−0.38 per 10% change, 95% CI −0.75 to −0.01, *P*=.04), so participants with lower stability of circadian activity rhythms had more severe symptoms. Heart rate variation between 4 AM and 6 AM was positively associated with PHQ-9 scores (β=0.90 per 10% change, 95% CI 0.04-1.78, *P*=.04), where a greater magnitude of heart rate variation indicated higher PHQ-9 scores. This digital biomarker correlated to depressive symptoms independent of sleep offset time, which might cause greater variation (Table S3 in Multimedia Appendix 1).

**Table 4:** Coefficients of multiple regression analysis for digital biomarkers and 9-item Patient Health Questionnaire (PHQ-9) scores. Fully adjusted models.

Predictor			β^a^ (95% CI)		*P* value
**Model 1**					
	Digital biomarker: IS.st.wd^b^	–2.974 (–5.202 to –0.747)		.009	
	Age	–0.114 (–0.163 to –0.064)		<.001	
	Gender (male)	–0.165 (–0.811 to 0.481)		.62	
	Ethnic group (Indian)	–0.297 (–1.393 to 0.798)		.59	
	Ethnic group (Malay)	–0.036 (–1.629 to 1.556)		.96	
	Ethnic group (others)	1.012 (–0.021 to 2.045)		.06	
	Marital status (single)	–0.633 (–1.341 to 0.076)		.08	
	Education level (university degree)	–1.023 (–2.079 to 0.034)		.06	
	Monthly income level (SGD 4000^c^ and above)	0.283 (–0.422 to 0.988)		.43	
	Alcohol consumption (yes)	–0.201 (–0.834 to 0.433)		.53	
	Smoking status (nonsmoker)	–0.768 (–2.075 to 0.54)		.25	
	Self-rated health (fair)	1.528 (–0.103 to 3.159)		.07	
	Self-rated health (good)	0.597 (–0.631 to 1.824)		.34	
	Self-rated health (very good)	–0.026 (–1.246 to 1.194)		.97	
	UCLA (University of California, Los Angeles) Loneliness Scale score	0.094 (0.062 to 0.127)		<.001	
	Sleep Hygiene Index (SHI) score	0.082 (0.022 to 0.143)		.008	
	Pittsburgh Sleep Quality Index (PSQI) score	0.362 (0.227 to 0.496)		<.001	
	Epworth Sleepiness Scale (ESS) score	0.06 (–0.014 to 0.134)		.11	
	Intercept	3.407 (–0.167 to 6.98)		.06	
**Model 2**					
	Digital biomarker: AC.st.15m.wd^d^	–3.843 (–7.567 to –0.119)		.04	
	Age	–0.113 (–0.163 to –0.063)		<.001	
	Gender (male)	–0.063 (–0.712 to 0.586)		.85	
	Ethnic group (Indian)	–0.19 (–1.285 to 0.904)		.73	
	Ethnic group (Malay)	–0.038 (–1.64 to 1.563)		.96	
	Ethnic group (others)	1.126 (0.09 to 2.162)		.03	
	Marital status (single)	–0.635 (–1.348 to 0.077)		.08	
	Education level (university degree)	–1.056 (–2.12 to 0.007)		.051	
	Monthly income level (SGD 4000 and above)	0.309 (–0.402 to 1.02)		.39	
	Alcohol consumption (yes)	–0.229 (–0.87 to 0.413)		.48	
	Smoking status (nonsmoker)	–0.741 (–2.056 to 0.573)		.27	
	Self-rated health (fair)	1.574 (–0.066 to 3.213)		.06	
	Self-rated health (good)	0.604 (–0.631 to 1.838)		.34	
	Self-rated health (very good)	–0.031 (–1.259 to 1.196)		.96	
	UCLA Loneliness Scale score	0.092 (0.059 to 0.124)		<.001	
	SHI score	0.091 (0.031 to 0.151)		.003	
	PSQI score	0.351 (0.216 to 0.486)		<.001	
	ESS score	0.062 (–0.013 to 0.136)		.11	
	Intercept	2.526 (–0.921 to 5.973)		.15	
**Model 3**					
	Digital biomarker: NHR.0406.cv^e^	9.096 (0.333 to 17.859)		.04	
	Age	–0.114 (–0.164 to –0.064)		<.001	
	Gender (male)	–0.246 (–0.909 to 0.416)		.47	
	Ethnic group (Indian)	–0.188 (–1.282 to 0.907)		.74	
	Ethnic group (Malay)	–0.193 (–1.802 to 1.415)		.81	
	Ethnic group (others)	0.985 (–0.058 to 2.028)		.06	
	Marital status (single)	–0.661 (–1.373 to 0.051)		.07	
	Education level (university degree)	–0.837 (–1.913 to 0.238)		.13	
	Monthly income level (SGD 4000 and above)	0.301 (–0.409 to 1.011)		.40	
	Alcohol consumption (yes)	–0.1 (–0.736 to 0.536)		.76	
	Smoking status (nonsmoker)	–0.72 (–2.035 to 0.595)		.28	
	Self-rated health (fair)	1.811 (0.166 to 3.456)		.03	
	Self-rated health (good)	0.763 (–0.479 to 2.005)		.23	
	Self-rated health (very good)	0.215 (–1.026 to 1.457)		.73	
	UCLA score	0.091 (0.059 to 0.124)		<.001	
	SHI score	0.103 (0.043 to 0.163)		.001	
	PSQI score	0.332 (0.195 to 0.47)		<.001	
	ESS score	0.061 (–0.013 to 0.135)		.11	
	Intercept	0.598 (–2.891 to 4.088)		.74	

^a^Unstandardized coefficients (β) with their 95% CIs and exact *P* values of digital biomarkers are reported as predictors of PHQ-9 scores in multiple regression models.

^b^IS.st.wd: steps-based interdaily stability on weekdays.

^c^A currency exchange rate of SGD 1=US $0.75 is applicable.

^d^AC.st.15m.wd: steps-based autocorrelation with weekday data aggregated into 15-minute intervals.

^e^NHR.0406.cv: coefficient of variation of nighttime heart rate between 4 AM and 6 AM.

### Depression Screening Using Digital Biomarkers and Machine Learning

The performance of the symptom severity prediction models was evaluated using the whole data set of 267 participants. The range of mean correlations between actual and predicted PHQ-9 scores across trained models was 0.14 to 0.27 (*R*^2^=0.03-0.08), the range of mean RMSE was 3.10 to 3.20, and the range of average MAE in holdout samples was 2.54 to 2.63 (Table 5, models A1-C1). Adding sociodemographic characteristics did not substantially improve performance (Table 5, models A2-C2). However, applying more conservative feature selection criteria, including digital biomarkers most correlated to the outcome, showed relatively better results compared to the less conservative criteria and including digital biomarkers less correlated to the outcome. The selected digital biomarkers are presented in Table 3 and listed in Table S4 in Multimedia Appendix 1.

**Table 5:** Performance of symptom-severity prediction model.

Model (feature set)	*R*^2^, mean (SD)	Pearson correlation, mean (SD)	Root mean square error, mean (SD)	Mean absolute error, mean (SD)
A1^a^	0.06 (0.04)	0.23 (0.09)	3.12 (0.22)	2.57 (0.15)
B1^b^	0.06 (0.04)	0.22 (0.09)	3.14 (0.22)	2.58 (0.15)
C1^c^	0.03 (0.03)	0.14 (0.08)	3.20 (0.21)	2.63 (0.14)
A2^d^	0.08 (0.05)	0.27 (0.09)	3.10 (0.22)	2.54 (0.16)
B2^d^	0.06 (0.04)	0.24 (0.09)	3.12 (0.22)	2.57 (0.15)
C2^d^	0.04 (0.03)	0.18 (0.08)	3.16 (0.20)	2.60 (0.13)

^a^Model A1 includes digital biomarkers selected at a significance level of <.01.

^b^Model B1 includes digital biomarkers selected at a significance level of <.05.

^c^Model C1 includes digital biomarkers selected at a significance level of <.05 and ≥.01.

^d^Models A2, B2, and C2 additionally include sociodemographic characteristics: age, gender, ethnic group, and marital status.

Next, we trained models for classification of the outcome of depression screening using different PHQ-9 score cutoff points to determining which participants were depressed or healthy. For the default cutoff point (ie, either baseline or follow-up PHQ-9 score of ≥10), the accuracy of the models in holdout folds was 86% (equal to the no information rate 86%), the sensitivity range was 3% to 13%, the specificity range was 98% to 100%, and the AUC range was 0.51 to 0.66 (Table 6, models A1-C1). Adding sociodemographic characteristics did not improve classification accuracy (Table 6, models A2-C2). However, applying more conservative feature selection criteria, including digital biomarkers most correlated to the outcome, showed relatively better results compared to the less conservative criteria and including digital biomarkers less correlated to the outcome.

For the second cutoff point option (ie, both baseline and follow-up PHQ-9 scores of ≥8), the accuracy of the models in holdout folds was 92% (no information ratio 92%), the sensitivity range was 0% to 5%, the specificity was 100%, and the AUC range was 0.54 to 0.67 (Table 6). Finally, for the third cutoff point option (ie, average PHQ-9 score of ≥8), the accuracy range of the models in holdout folds was 85% to 87% (no information ratio 84%), the sensitivity range was 2% to 24%, the specificity range was 97% to 100%, and the AUC range was 0.62 to 0.74 (Table 6). Thus, the performance of models classifying the outcome of depression screening was relatively better when the third cutoff point was applied.

In general, the accuracy of models predicting severity of depressive symptoms and depression screening status based on digital biomarkers in the whole sample was poor, probably indicating a significant heterogeneity of data within groups of depressed and healthy participants.

**Table 6:** Performance of the prediction models of depression screening status for different cutoff points and varying sets of selected digital biomarkers.

Outcome cutoff point and model (feature set)		No information rate	Accuracy	Sensitivity	Specificity	PPV^a^	NPV^b^	κ	AUC^c^
**PHQ-9^d^ score of ≥10 at assessment 1 or 2 (n=38 in depressed group)**									
	A1^e^	0.86	0.86	0.05	0.99	0.50	0.86	0.07	0.66
	B1^f^	0.86	0.86	0.03	1.00	0.50	0.86	0.04	0.63
	C1^g^	0.86	0.86	0.03	1.00	1.00	0.86	0.04	0.51
	A2^h^	0.86	0.86	0.03	1.00	0.50	0.86	0.04	0.64
	B2^h^	0.86	0.86	0.13	0.98	0.56	0.87	0.17	0.64
	C2^h^	0.86	0.86	0.05	1.00	0.67	0.86	0.08	0.61
**PHQ-9 score of ≥8 at assessment 1 or 2 (n=22 in depressed group)**									
	A1	0.92	0.92	0.05	1.00	1.00	0.92	0.08	0.64
	B1	0.92	0.92	0.00	1.00	N/A^i^	0.92	0.00	0.54
	C1	0.92	0.92	0.05	1.00	1.00	0.92	0.08	0.56
	A2	0.92	0.92	0.05	1.00	1.00	0.92	0.08	0.67
	B2	0.92	0.92	0.00	1.00	N/A	0.92	0.00	0.58
	C2	0.92	0.92	0.05	1.00	1.00	0.92	0.08	0.62
**PHQ-9 average score of ≥8 (n=42 in depressed group)**									
	A1	0.84	0.85	0.12	0.99	0.63	0.86	0.16	0.70
	B1	0.84	0.85	0.19	0.97	0.57	0.87	0.23	0.67
	C1	0.84	0.85	0.02	1.00	1.00	0.85	0.04	0.62
	A2	0.84	0.87	0.24	0.99	0.77	0.87	0.31	0.74
	B2	0.84	0.85	0.17	0.98	0.64	0.86	0.21	0.70
	C2	0.84	0.85	0.07	1.00	1.00	0.85	0.12	0.62

^a^PPV: positive predictive value.

^b^NPV: negative predictive value.

^c^AUC: area under the curve.

^d^PHQ-9: 9-item Patient Health Questionnaire.

^e^Model A1 includes digital biomarkers selected at a significance level of <.01.

^f^Model B1 includes digital biomarkers selected at a significance level of <.05.

^g^Model C1 includes digital biomarkers selected at a significance level of <.05 and ≥.01.

^h^Models A2, B2, and C2 additionally include sociodemographic characteristics: age, gender, ethnic group, and marital status.

^i^N/A: not applicable, due to division by zero.

### Detecting Individuals at High Risk of Depression Against Those With No Risk

We retrained models using random downsampling of healthy participants from the lowest range of PHQ-9 scores (0-4) to address the class imbalance in our data and to increase the contrast between compared groups, similar to Sano et al [39]. We believe that excluding participants with midrange (ie, borderline) PHQ-9 scores results in the larger difference between groups and, therefore, increases the discriminatory power of digital biomarkers. We excluded participants with zero scores (n=24) due to concerns in honesty of their responses to minimize the bias in self-reported outcome. We used three contrasted subsamples varying by the PHQ-9 cutoff points determining the depressed group similar to the classification in the whole sample; additionally, we used the contrasted subsample comprised of the top 20% and the bottom 20% of participants by average PHQ-9 scores. For the default cutoff point (ie, either baseline or follow-up PHQ-9 score of ≥10), the contrasted sample included 78 participants (38 depressed and 40 healthy); for the second cutoff point (ie, both baseline and follow-up PHQ-9 scores of ≥8), the contrasted sample included 44 participants (22 depressed and 22 healthy); and for the third cutoff point (ie, average PHQ-9 scores of ≥8), the contrasted sample included 84 participants (42 depressed and 42 healthy). Finally, the fourth subsample included 96 participants (48 depressed and 48 healthy), where the range of PHQ-9 scores for the healthy group was 0.5 to 1.5 and for the depressed group was 7.5 to 14. Subsamples mainly did not differ from the whole sample in terms of sociodemographic characteristics, with two exceptions: the second subsample had a slightly younger age, and the fourth subsample did not have a gender bias (see Tables S5 to S8 in Multimedia Appendix 1).

Importantly, feature selection for these models was done using the entire sample (ie, selected digital biomarkers were based on statistical associations found in the entire sample, not in subsamples, and remained the same for all models). Otherwise, if feature selection is done each time for a new subsample with a different cutoff point, sets of digital biomarkers would be arbitrary and would vary depending on a particular sample composition. This allows us to mitigate overgeneralization that is possible in skewed samples, because models were trained with digital biomarkers inferred from the entire sample. For performance evaluation of these models, we used stratified repeated cross-validation with 4 folds and 25 repeats.

**Figure 3:**
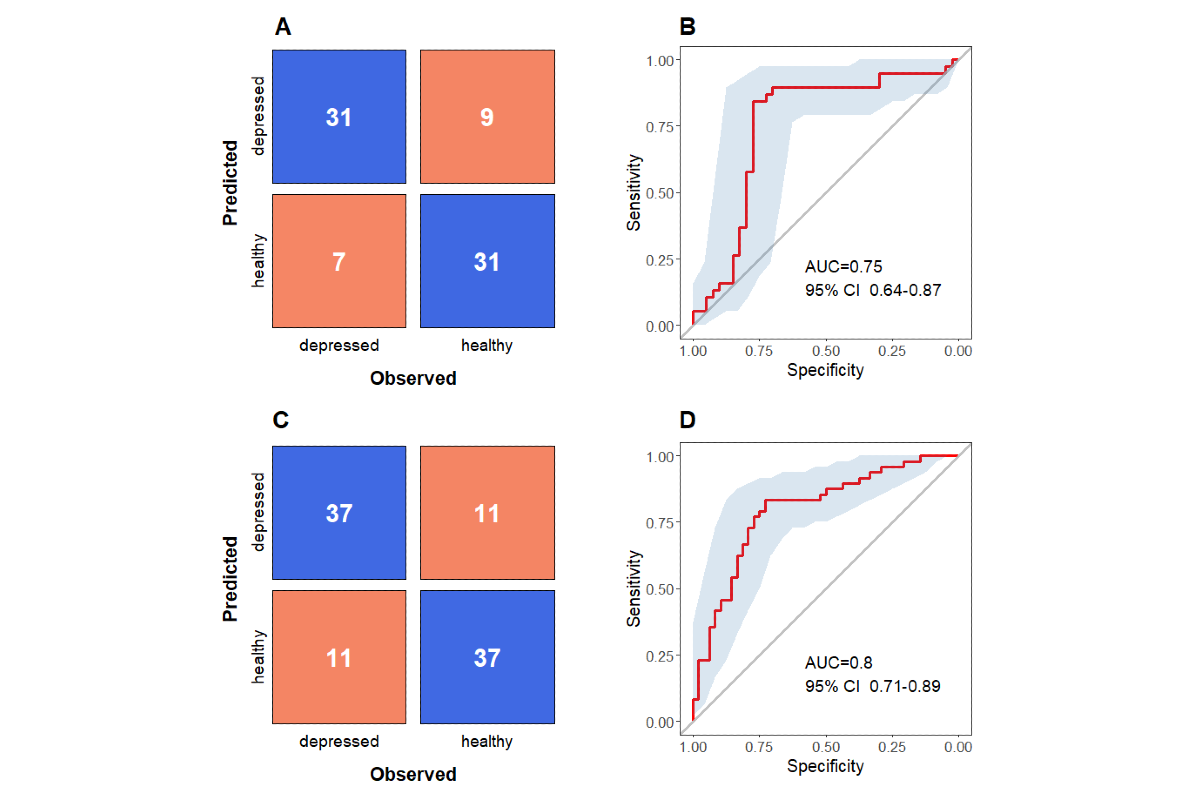
A and B. Performance evaluation of model A based on the contrasted subsample with default cutoff point (ie, either baseline or follow-up PHQ-9 score of ≥10). A. Confusion matrix of predicted and observed outcomes. B. Area under the curve (AUC) with 95% CI. C and D. Performance evaluation of model A based on the contrasted subsample comprised of the top 20% and bottom 20% of participants by average PHQ-9 score. C. Confusion matrix of predicted and observed outcomes. D. AUC with 95% CI. PHQ-9: 9-item Patient Health Questionnaire.

Similar to the previous step of modeling, using more conservative feature selection criteria consistently resulted in better performance compared to the less conservative criteria. The best model was based on the contrasted subsample with default cutoff point (ie, either baseline or follow-up PHQ-9 score of ≥10) and has correctly predicted the depression screening status in 80% of participants from holdout folds, with a sensitivity of 82% and a specificity of 78% (Table 7 and Figure 3, A and B). Alternatively, the model based on the subsample comprised of the top 20% and bottom 20% of participants by average PHQ-9 score achieved the highest AUC of 0.8, and had accuracy, sensitivity, and specificity values of 77% each (Table 7 and Figure 3, C and D). Additionally, using the contrasted subsample with the top 20% and bottom 20% of participants, we trained models without statistical feature selection but with different feature subsets varying by categories (ie, activity metrics, nonparametric circadian rhythm metrics, cosinor-based metrics, heart rate metrics, and sleep metrics). Among these models, the model with heart rate metrics had the best accuracy, sensitivity, and AUC—70%, 71%, and 72%, respectively—and the model with nonparametric circadian rhythm metrics had the best specificity of 76% (see Table S9 in Multimedia Appendix 1); however, models with correlation-based feature selection outperformed all of these models.

**Figure 4:**
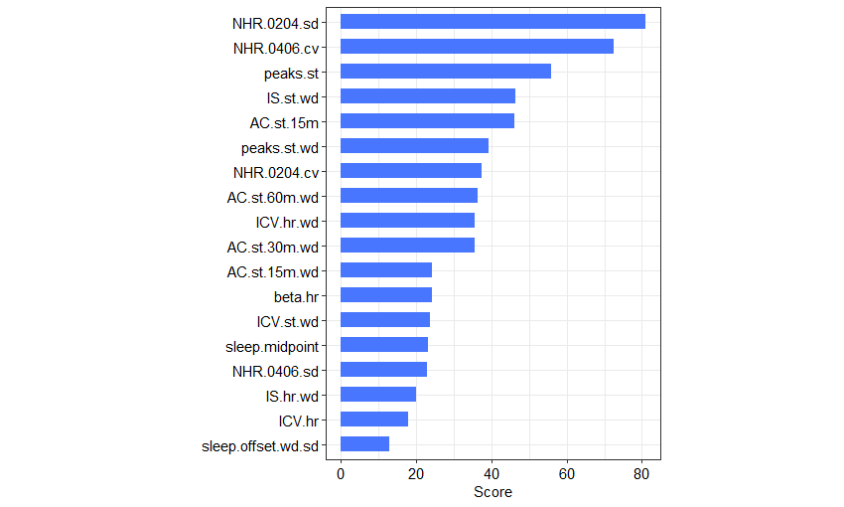
Relative importance of digital biomarkers averaged from four models. 15m, 30m, and 60m: 15- , 30-, and 60-minute time interval in which raw data were aggregated; AC: autocorrelation; cv: coefficient of variation; hr: heart rate based; ICV: interdaily coefficient of variation; IS: interdaily stability; NHR: nighttime heart rate in a specified 2-hour time interval (0204: 2 AM-4 AM; 0406: 4 AM-6 AM); sd: standard deviation; st: steps based; wd: weekdays based.

**Figure 5:**
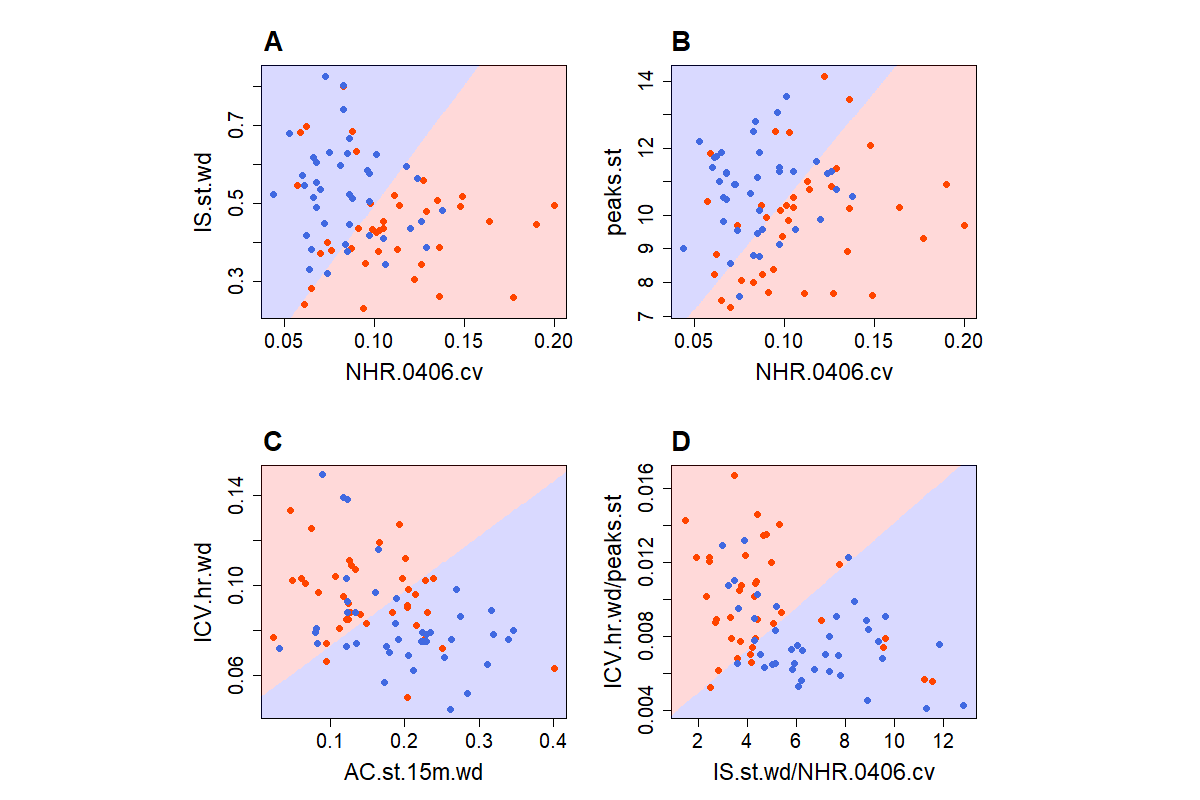
Digital biomarkers of depressed and healthy participants. All scatterplots show the contrasted subsample with the default cutoff point. Red dots represent depressed participants and blue dots represent healthy participants; the background coloring represents a decision boundary of linear discriminant analysis. A. IS.st.wd: weekday steps–based interdaily stability; NHR.0406.cv: variation of heart rate between 4 AM and 6 AM. B. peaks.st: daily steps–based peaks; NHR.0406.cv: variation of heart rate between 4 AM and 6 AM. C. ICV.hr.wd: interdaily coefficient of variation of heart rate on weekdays; AC.st.15m.wd: autocorrelation of weekday steps–based rhythm (steps aggregated in 15-minute intervals). D. ICV.hr.wd/peaks.st: interdaily coefficient of variation of heart rate on weekdays divided by daily steps–based peaks; IS.st.wd/NHR.0406.cv: weekday steps–based interdaily stability divided by variation of heart rate between 4 AM and 6AM.

Relative importance of digital biomarkers was extracted and averaged from the best models (model A from Table 7) for each of four contrasted subsamples (Figure 4). The scatterplots in Figure 5 illustrate how different combinations of digital biomarkers can discriminate between provisionally depressed and healthy participants in the contrasted sample.

**Table 7:** Performance of the prediction of depression screening status in contrasted subsamples.

Contrasted subsample and model (feature set)			No information rate		Accuracy		Sensitivity		Specificity		PPV^a^		NPV^b^		κ		AUC^c^
**PHQ-9^d^ score of ≥10 at assessment 1 or 2 (n=78)**																	
	A^e^	0.51		0.80		0.82		0.78		0.78		0.82		0.59		0.75	
	B^f^	0.51		0.77		0.79		0.75		0.75		0.79		0.54		0.74	
	C^g^	0.51		0.71		0.74		0.68		0.68		0.73		0.41		0.70	
**PHQ-9 score of ≥8 at assessment 1 or 2 (n=44)**																	
	A	0.50		0.71		0.73		0.68		0.70		0.71		0.41		0.71	
	B	0.50		0.71		0.77		0.64		0.68		0.74		0.41		0.68	
	C	0.50		0.71		0.73		0.68		0.70		0.71		0.41		0.64	
**PHQ-9 average score of ≥8 (n=84)**																	
	A	0.50		0.77		0.79		0.76		0.77		0.78		0.55		0.76	
	B	0.50		0.71		0.71		0.71		0.71		0.71		0.43		0.71	
	C	0.50		0.61		0.64		0.57		0.60		0.62		0.21		0.57	
**Top 20% and bottom 20% by average PHQ-9 score (n=96)**																	
	A	0.50		0.77		0.77		0.77		0.77		0.77		0.54		0.80	
	B	0.50		0.74		0.73		0.75		0.75		0.74		0.48		0.76	
	C	0.50		0.65		0.63		0.67		0.65		0.64		0.29		0.62	

^a^PPV: positive predictive value.

^b^NPV: negative predictive value.

^c^AUC: area under the curve.

^d^PHQ-9: 9-item Patient Health Questionnaire.

^e^Model A includes digital biomarkers selected at a significance level of <.01.

^f^Model B includes digital biomarkers selected at a significance level of <.05.

^g^Model C includes digital biomarkers selected at a significance level of <.05 and ≥.01.

## Discussion

### Principal Findings

In this study, using statistical analysis and machine learning, we demonstrated that some known and novel digital biomarkers based on behavioral and physiological data from consumer wearables could indicate increased risk of depression in a multiethnic working population. We found that greater severity of depressive symptoms was robustly associated with greater variation of nighttime heart rate between 4 AM and 6 AM; it was also associated with lower regularity of weekday circadian activity rhythms based on steps and measured with nonparametric measures of IS and autocorrelation. Effects of these digital biomarkers on symptom severity were stable and independent of all confounders including strong predictors of depression, such as sleep quality and loneliness. Additionally, we found that lower regularity of heart rate circadian rhythm, fewer steps-based daily peaks, greater steepness of the heart rate rhythm curve, later sleep midpoint, and greater variation of sleep offset time were associated with a greater severity of depressive symptoms, yet these associations were less reliable and became nonsignificant in regression models with covariates. Despite several reliable associations, our evidence showed limited ability of digital biomarkers to detect depression in the whole sample of working adults. However, in balanced and contrasted subsamples comprised of provisionally depressed participants and healthy participants with no risk of depression, the model achieved an accuracy of 80%, a sensitivity of 82%, and a specificity of 78% in detecting subjects at high risk of depression. Similar performance has been achieved across all models trained using alternative contrasted subsamples. Thus, predictive models based on a combination of these digital biomarkers could quite accurately discriminate individuals with a high risk of depression from individuals with no risk.

### Comparison With Previous Research

We compared our study to research investigating relationships between specific actigraphy metrics and depressive disorder. Firstly, regarding circadian rhythm metrics, we found that a lower steps-based weekday IS—a nonparametric measure of rhythm regularity—was robustly associated with a greater severity of depressive symptoms independent of confounders, supporting and extending existing evidence [31,60], including one large-sample study [34]. However, there are several studies that did not find IS to be related to depressive symptoms or other mental disorders [27,35,36]. In support of this association, we also found steps-based autocorrelation during weekdays—a complementary and alternative nonparametric measure of rhythm regularity—to be robustly correlated to symptom severity independent of all confounders. Rhythm stability metrics based on heart rate were also correlated to severity of depressive symptoms, yet these associations became nonsignificant in fully adjusted regression models. Likewise, among cosinor-based metrics, our data indicated lower pseudo-*F* statistic values and later acrophase, both based on steps and heart rate, in participants with more severe symptoms, which is consistent with existing evidence [22,23,25,26,29,30], yet these correlations became nonsignificant after the FDR correction and in multiple regression analysis. Perhaps this supports the advantage of nonparametric indicators over cosinor-based metrics, where the former appear more robust and sensitive to indicate depressive symptomatology in nonclinical samples. In general, results of our study together with previous evidence demonstrated that people with more severe depressive symptoms tended to have less stable circadian activity rhythms.

There are several key novel approaches regarding circadian rhythm analysis in our study. First, we used step counts and heart rate data from consumer wearables instead of “activity counts”—a measure of total linear acceleration—from research-grade devices commonly used in other studies. There are few studies that used alternative source data for circadian rhythm analysis; for example, heart rate [32] or skin temperature [36]. Second, we analyzed weekday circadian rhythms separately that were found to be stronger predictors rather than rhythms based on all days. Although weekday rhythm is mainly determined by work routine, the ability to adherently follow this routine better discriminates between depressed and healthy individuals, where healthy people demonstrated a greater regularity. Third, we showed the value of novel rhythm stability metrics—autocorrelation and ICV—as risk markers of depression. Finally, we first showed that a greater severity of depressive symptoms was associated with a fewer steps-based daily peaks, which perhaps reflects fewer distinct activities happening over a day, indicating a diagnostic symptom of anhedonia (ie, loss of interest in activities).

Secondly, our findings suggest that a greater variation of nighttime heart rate between 2 AM and 4 AM and between 4 AM and 6 AM indicates greater severity of depressive symptoms, which is aligned with previous electrocardiogram research that showed that changes in heart rate during sleep may be a valid physiological marker of depression [56,61]. However, study participants were recruited from a sleep disorder clinic and had sleep complaints apart from diagnosed depressive disorder [56].

Thirdly, in contrast to some previous findings [15-17,19,28,33,62], our data did not show reduced levels of locomotor activity in depressed participants in terms of less time spent in moderate to vigorous physical activity, fewer daily steps, or more sedentary time. This might be due to the overestimation of the time spent in high-intensity activities by consumer wearables [42] and due to the overall high level of physical activity in the Singapore population [63], where low physical activity may be a rare depression risk marker. In addition, our data did not show a relationship with cosinor-based metrics estimating the level of activity, including mesor and rhythm amplitude [24,25], or with nonparametric measures, including M10, L5, and RA.

Finally, the analysis of sleep data showed that later sleep midpoint and offset time were associated with more severe depressive symptoms, which is consistent with the existing evidence [16,20,22,23]; however, we did not find that shortened sleep duration, increased SOL, lower SE, and longer WASO were related to more severe symptoms, contributing to the mixed results from previous actigraphy studies [16,22-24,34]. This discrepancy may be explained by the lack of participants with clinical depression in our sample or by the limited accuracy of Fitbit wearables in measuring sleep compared to PSG [45,46].

The results of our study are also comparable to a few previous studies that used machine learning with wearable sensor data for depression detection. Jacobson et al achieved a high accuracy in detecting depressed individuals, but their approach has some limitations [37]. First, although their model classified clinically diagnosed patients and healthy controls, 5 out of 23 depressed patients in their sample were hospitalized, which significantly limits generalizability of their actigraphy data–based model. Second, they extracted and explored thousands of features which, without correction for multiple comparisons, by chance might correlate to the outcome variable in the given sample but may not in other samples; therefore, it is highly likely that significant digital biomarkers will be inconsistent across different samples. Third, the number of spectral analysis–based features depended on the minimum duration of actigraphy data available across participants; therefore, some features are not universal and would be unavailable for a shorter observation period. Finally, most features were extracted mechanically without relying on domain knowledge or previous findings and remained uninterpreted. For example, interpretation of spectral density features, which were the only important predictors in their model, remained unclear. Contrary to this study, our approach relies on interpretable digital biomarkers and meaningful behavioral and physiological phenomena underlying these markers.

In another study, Tazawa at al achieved an accuracy of 76% in detection of depressed individuals based on 236 assessments of 85 participants, which is very similar to the performance of our models trained with contrasted subsamples [33]. Although our best model had 9% higher sensitivity (82% vs 73%), which is more important than higher specificity if using these models for passive screening to address underdiagnosis of depression, the direct comparison between studies is problematic due to the specific downsampling used in our models. In addition, there are important differences between studies in both available sensor data and outcome measurement. First, Tazawa and colleagues used the Hamilton Depression Rating Scale for symptom assessment, and their sample included patients with clinical depression. Second, they had more types of sensor data, including skin temperature, which were the most indicative of depression. Finally, they mainly used distribution characteristics of the per-hour data and correlations between different data types as digital biomarkers but did not use circadian rhythm metrics.

It is worth mentioning the study by Sano et al, whose model based on wearable sensor data achieved a comparable classification accuracy of 87% [39]. The important methodological similarity between the studies is that Sano et al similarly used a contrasted subsample for training of the models, yet theirs was comprised of an even smaller fraction and number of participants (ie, top 12% and bottom 12% of participants; n=47). Despite methodological similarities, the studies are different in terms of population, outcome measurement, and extraction of digital biomarkers. They studied college students and used the mental component summary score from the 12-item Short Form Health Survey for mental well-being assessment, which was not intended to screen for depression, unlike the PHQ-9 used in our study. Furthermore, they collected skin conductivity and skin temperature data in addition to accelerometer data and used data distribution characteristics (eg, mean and median) at different times of day as digital biomarkers, but they did not analyze and harness circadian rhythm metrics. Overall, the key novel approach in our study, as compared to existing efforts, was the use of data from widespread consumer wearables and the use of circadian rhythm metrics as digital biomarkers in predictive modeling with machine learning.

### Possible Mechanisms

Regularity of circadian rhythm and variation of nighttime heart rate were the most robust digital biomarkers; below, we outline possible psychosocial and neurobiological mechanisms linking them to depressive disorder. The relationships between depression and circadian rhythms in behavior and physiology are probably bidirectional, but underlying neurobiological mechanisms remain unknown [64,65]. Existing evidence shows that disturbed rest-activity rhythms, as, for example, in shift workers, lead to desynchronization of internal molecular clocks, thereby disturbing circadian biochemical processes, secretion of hormones, metabolic functions, and physiological parameters [66-68]. In turn, disturbed master clocks at the molecular level could lead to neurobiological dysfunction that may generate depressive mood [69]. It has been documented that patients with major depression have elevated nocturnal body temperature, increased cortisol, lower melatonin, and lower norepinephrine levels [70,71]. On the other hand, mood disorders affect circadian activity rhythms through psycho-cognitive pathways: a depressed individual can experience increased apathy, impaired deliberative cognitive control, greater impulsivity, and other affects, which may result in inconsistent behavior, disturbed routine, and disturbed circadian rhythms. We may speculate that observed associations either support the social rhythm hypothesis or probably capture nuanced behavioral manifestations of depressive symptomatology [72-74]. Regarding the variation of heart rate in nighttime intervals, this digital biomarker could indicate depressive disorder because an increased arousal of autonomic nervous system that is possible with depression is likely to affect heart rate dynamics during sleep [56].

### Strengths and Limitations

Strengths of our study include a relatively long period of continuous sensing and activity tracking in free-living settings, a relatively large workplace-based sample, use of correction for multiple testing in statistical analysis, use of a wide range of covariates for model adjustments in regression analysis, and use of cross-validation with multiple resampling in machine learning modeling. Moreover, as digital biomarkers, we used only metrics that meaningfully characterize everyday behavior and human physiology relevant to depressive disorder, avoiding extraction of a multitude uninterpretable features and black box approaches.

This study also has several limitations. First, we studied working adults who represent the generally healthy population and had not been diagnosed with depression. The absence of participants with clinical depression might cause a lack of contrast in behavioral and physiological data between depressed and healthy participants, which impedes discovery of reliable digital biomarkers. In addition, we used the PHQ-9 for depression screening, which is a self-reported scale with limited accuracy. Second, our participants were mostly highly educated university employees with sedentary jobs who might have specific psycho-behavioral characteristics. Third, the cross-sectional design of the study does not allow causal inferences. Fourth, the poor predictability of depressive symptomatology in the whole sample highlights a possible limitation, in principle, of using this set of digital biomarkers alone for depression screening universally due to prominent interindividual differences and heterogeneity that appear in naturalistic settings [36]. Finally, predictive models were retrained using balanced and contrasted subsamples equally comprised of depressed participants and healthy participants with no risk of depression; therefore, they probably suffer from overfitting and would perform worse on new samples. However, the set of selected digital biomarkers used in these models remained the same as in models with the full data set.

### Conclusions and Future Research

Further discovery of digital biomarkers from wearable sensors has the potential to facilitate early, unobtrusive, continuous, and cost-effective detection of depression in the general population. This study showed that some known and novel digital biomarkers based on data from consumer wearables could indicate increased risk of depression in the working population. The predictive model based on a combination of these digital biomarkers could discriminate individuals with high risk of depression from individuals with no risk. Further research should examine and validate digital biomarkers with longitudinal design, because dynamic changes and deviations from a baseline are more likely to indicate risk of depression rather than one-time snapshots. Second, although the idea of inferring universal digital biomarkers is very tempting, the development of semipersonalized models adjusted for differential baseline characteristics can bring more accurate and clinically relevant predictions. Third, wearable sensor data can be enriched with smartphone data, which will enable more comprehensive digital phenotyping for depression detection [39,75].

## Appendix

Multimedia Appendix 1 Additional tables with statistics.

Multimedia Appendix 2 Digital biomarkers.

Multimedia Appendix 3 R-code scripts for data processing and statistical analysis.

Multimedia Appendix 4 Participant flowchart. HR: heart rate.

## Abbreviations

AUC: area under the curve
bpm: beats per minute
CV: coefficient of variation
DART: Dropouts meet Multiple Additive Regression Trees
FDR: false discovery rate
ICV: interdaily coefficient of variation
IS: interdaily stability
IV: intradaily variability
L2NIC: Land and Liveability National Innovation Challenge
L5: mean value of the 5 consecutive least active hours of the day
M10: mean activity of the 10 consecutive most active hours of the day
MAE: mean absolute error
MET: metabolic equivalent
NTU: Nanyang Technological University
PHQ-9: 9-item Patient Health Questionnaire
PSG: polysomnography
RA: relative amplitude
REDCap: Research Electronic Data Capture
REM: rapid eye movement
RHR: resting heart rate
RMSE: root mean square error
RMSSD: root mean square of successive differences
SE: sleep efficiency
SOL: sleep onset latency
TST: total sleep time
UCLA: University of California, Los Angeles
WASO: wake after sleep onset
