# Supplementary material for: Digital Biomarkers for Depression Screening With Wearable Devices: Cross-sectional Study With Machine Learning Modeling
Source: JMIR Mhealth Uhealth. 2021 Oct 25;9(10):e24872. doi: 10.2196/24872 (PMC8576601; doi:10.2196/24872)
Supplement: Multimedia Appendix 1 [file mhealth_v9i10e24872_app1.pdf]

Multimedia Appendix 1. Additional tables with statistics

Table S1. The number of complete days and the corresponding number of participants

Complete days	N participants
0	2
3	2
6	2
7	1
8	2
9	4
10	6
11	15
12	22
13	56
14	85
15	33
16	24
17	13
18	6
19	9
20	6
21	2

Table S2. Coefficients and associated 95% confidence interval (95% CI) of multiple regression analysis for digital biomarkers and PHQ-9 score. Model adjusted for sociodemographic covariates.

Predictor	Beta	95% CI	*P* value
**Model 1**			
Digital biomarker – IS.st.wd	-3.649	(-6.483 to -0.816)	.012
Age	-0.106	(-0.167 to -0.045)	.001
Gender (male)	-0.707	(-1.489 to 0.075)	.076
Ethnic group (Indian)	0.47	(-0.925 to 1.865)	.507
Ethnic group (Malay)	1.997	(0.017 to 3.977)	.048
Ethnic group (Others)	0.861	(-0.452 to 2.175)	.198
Marital status (single)	0.188	(-0.709 to 1.085)	.681
Education level (university degree)	-1.199	(-2.541 to 0.143)	.08
Monthly income level (SGD 4,000 and above)	0.149	(-0.755 to 1.054)	.745
Intercept	10.153	(6.917 to 13.388)	<.001
**Model 2**			
Digital biomarker – AC.st.15m.wd	-5.79	(-10.523 to -1.057)	.017
Age	-0.107	(-0.168 to -0.046)	.001
Gender (male)	-0.587	(-1.37 to 0.196)	.141
Ethnic group (Indian)	0.571	(-0.818 to 1.959)	.419
Ethnic group (Malay)	1.976	(-0.006 to 3.958)	.051
Ethnic group (Others)	0.986	(-0.322 to 2.295)	.139
Marital status (single)	0.199	(-0.699 to 1.097)	.663
Education level (university degree)	-1.25	(-2.595 to 0.095)	.068
Monthly income level (SGD 4,000 and above)	0.207	(-0.701 to 1.115)	.654
Intercept	9.353	(6.322 to 12.384)	<.001
**Model 3**			
Digital biomarker – AC.st.30m.wd	-4.355	(-8.041 to -0.668)	.021
Age	-0.105	(-0.167 to -0.044)	.001
Gender (male)	-0.679	(-1.462 to 0.103)	.089
Ethnic group (Indian)	0.571	(-0.819 to 1.961)	.419
Ethnic group (Malay)	2.001	(0.017 to 3.984)	.048
Ethnic group (Others)	0.878	(-0.438 to 2.193)	.19
Marital status (single)	0.178	(-0.721 to 1.077)	.697
Education level (university degree)	-1.181	(-2.526 to 0.163)	.085
Monthly income level (SGD 4,000 and above)	0.193	(-0.715 to 1.101)	.675
Intercept	9.408	(6.354 to 12.463)	<.001
**Model 4**			
Digital biomarker – NHR.0204.sd	0.174	(0.013 to 0.334)	.034
Age	-0.097	(-0.159 to -0.036)	.002
Gender (male)	-0.748	(-1.537 to 0.041)	.063
Ethnic group (Indian)	0.624	(-0.765 to 2.013)	.377
Ethnic group (Malay)	1.775	(-0.218 to 3.768)	.081
Ethnic group (Others)	0.917	(-0.398 to 2.233)	.171
Marital status (single)	0.155	(-0.747 to 1.057)	.736
Education level (university degree)	-0.873	(-2.249 to 0.502)	.212
Monthly income level (SGD 4,000 and above)	0.082	(-0.826 to 0.99)	.859
Intercept	9.408	(6.354 to 12.463)	<.001
**Model 5**			
Digital biomarker – NHR.0204.cv	10.898	(0.251 to 21.544)	.045

Age	-0.096	(-0.158 to -0.034)	.003
Gender (male)	-0.805	(-1.604 to -0.006)	.048
Ethnic group (Indian)	0.694	(-0.694 to 2.082)	.325
Ethnic group (Malay)	1.785	(-0.211 to 3.78)	.079
Ethnic group (Others)	0.943	(-0.372 to 2.259)	.159
Marital status (single)	0.183	(-0.718 to 1.085)	.689
Education level (university degree)	-0.882	(-2.26 to 0.496)	.209
Monthly income level (SGD 4,000 and above)	0.079	(-0.83 to 0.988)	.864
Intercept	6.789	(3.503 to 10.075)	<.001
**Model 6**			
Digital biomarker – NHR.0406.sd	0.259	(0.09 to 0.427)	.003
Age	-0.121	(-0.183 to -0.06)	<.001
Gender (male)	-0.751	(-1.53 to 0.029)	.059
Ethnic group (Indian)	0.368	(-1.025 to 1.761)	.604
Ethnic group (Malay)	1.497	(-0.494 to 3.489)	.14
Ethnic group (Others)	0.808	(-0.5 to 2.116)	.225
Marital status (single)	0.185	(-0.707 to 1.078)	.683
Education level (university degree)	-0.864	(-2.214 to 0.487)	.209
Monthly income level (SGD 4,000 and above)	0.267	(-0.638 to 1.171)	.562
Intercept	7.086	(4.081 to 10.09)	<.001
**Model 7**			
Digital biomarker – NHR.0406.cv	16.601	(5.656 to 27.547)	.003
Age	-0.116	(-0.177 to -0.055)	<.001
Gender (male)	-0.856	(-1.645 to -0.067)	.034
Ethnic group (Indian)	0.444	(-0.943 to 1.831)	.529
Ethnic group (Malay)	1.491	(-0.502 to 3.485)	.142
Ethnic group (Others)	0.812	(-0.496 to 2.121)	.223
Marital status (single)	0.212	(-0.68 to 1.105)	.64
Education level (university degree)	-0.883	(-2.233 to 0.466)	.199
Monthly income level (SGD 4,000 and above)	0.254	(-0.65 to 1.158)	.58
Intercept	6.866	(3.814 to 9.917)	<.001
**Model 8**			
Digital biomarker – IS.hr.wd	-4.457	(-7.468 to -1.447)	.004
Age	-0.108	(-0.169 to -0.047)	.001
Gender (male)	-0.712	(-1.49 to 0.067)	.073
Ethnic group (Indian)	0.182	(-1.241 to 1.605)	.801
Ethnic group (Malay)	1.766	(-0.209 to 3.742)	.079
Ethnic group (Others)	0.812	(-0.498 to 2.122)	.223
Marital status (single)	0.122	(-0.774 to 1.018)	.789
Education level (university degree)	-0.987	(-2.33 to 0.356)	.149
Monthly income level (SGD 4,000 and above)	0.183	(-0.719 to 1.084)	.69
Intercept	11.333	(7.795 to 14.87)	<.001
**Model 9**			
Digital biomarker – ICV.hr.wd	20.884	(1.123 to 40.645)	.038
Age	-0.096	(-0.158 to -0.034)	.003
Gender (male)	-0.845	(-1.651 to -0.04)	.04
Ethnic group (Indian)	0.443	(-0.968 to 1.854)	.537
Ethnic group (Malay)	1.762	(-0.233 to 3.758)	.083
Ethnic group (Others)	0.855	(-0.468 to 2.178)	.204
Marital status (single)	0.141	(-0.762 to 1.045)	.759
Education level (university degree)	-1.005	(-2.362 to 0.352)	.146

Monthly income level (SGD 4,000 and above)	0.149	(-0.759 to 1.057)	.747
Intercept	6.159	(2.589 to 9.728)	.001
**Model 10**			
Digital biomarker – AC.hr.60m.wd	-3.56	(-6.716 to -0.405)	.027
Age	-0.107	(-0.169 to -0.046)	.001
Gender (male)	-0.738	(-1.525 to 0.049)	.066
Ethnic group (Indian)	0.296	(-1.139 to 1.731)	.685
Ethnic group (Malay)	1.8	(-0.19 to 3.789)	.076
Ethnic group (Others)	0.91	(-0.404 to 2.225)	.174
Marital status (single)	0.182	(-0.718 to 1.082)	.691
Education level (university degree)	-1.021	(-2.373 to 0.332)	.139
Monthly income level (SGD 4,000 and above)	0.199	(-0.71 to 1.108)	.667
Intercept	10.251	(6.872 to 13.629)	<.001
**Model 11**			
Digital biomarker – AC.hr.30m.wd	-3.792	(-7.111 to -0.472)	.025
Age	-0.109	(-0.17 to -0.048)	.001
Gender (male)	-0.729	(-1.515 to 0.057)	.069
Ethnic group (Indian)	0.327	(-1.099 to 1.754)	.652
Ethnic group (Malay)	1.798	(-0.192 to 3.787)	.076
Ethnic group (Others)	0.905	(-0.409 to 2.22)	.176
Marital status (single)	0.198	(-0.701 to 1.098)	.664
Education level (university degree)	-1.036	(-2.387 to 0.315)	.132
Monthly income level (SGD 4,000 and above)	0.212	(-0.698 to 1.122)	.647
Intercept	10.191	(6.85 to 13.533)	<.001

The table shows unstandardized coefficients (Beta), 95% confidence intervals and exact *P* values of digital biomarkers as predictors of PHQ-9 in multiple regression models adjusted for sociodemographic covaraites. Abbreviations: CI – confidence interval; NHR.0406.cv – coefficient of variation of night-time heart rate between 4:00–6:00 am, IS.st.wd – steps-based inter-daily stability on weekdays, IS.hr.wd – heart rate-based inter-daily stability on weekdays, ICV.st.wd – steps-based inter-daily coefficient of variation on weekdays, AC.st.15m.wd – steps-based autocorrelation with weekday data aggregated into 15-minute intervals.

Table S3. Multiple regression analysis for night-time variation of HR and sleep offset time

Predictors	Dependent variable: PHQ-9 score	
	β (95% CI)	*P* value
NHR.0406.cv	19.35 (8.79, 29.91)	<0.001
sleep.offset.h	0.02 (-0.35, 0.38)	0.94
Age	-0.11 (-0.16, -0.06)	<0.001
Gender (male)	-0.94 (-1.71, -0.17)	0.02
Constant	5.76 (1.61, 9.91)	0.007

The table shows unstandardized coefficients (β), confidence intervals and exact *P* values of digital biomarkers as predictors of PHQ-9 in multiple regression model. The model was adjusted for age and gender. Abbreviations: CI – confidence interval; NHR.0406.cv – coefficient of variation of night-time heart rate between 4:00–6:00 am, sleep.offset.h – average sleep offset time.

Table S4. The list of digital biomarkers used in prediction modeling with machine learning according to selection criteria with different levels of significance

Category	Digital biomarker	*P* value category
Circadian rhythm – nonparametric	AC.st.15m<br>AC.st.15m.wd<br>AC.st.30m.wd<br>AC.st.60m.wd<br>ICV.hr<br>ICV.hr.wd<br>ICV.st.wd<br>IS.hr.wd<br>IS.st.wd<br>peaks.st<br>peaks.st.wd	< .01
Circadian rhythm – cosinor-based	beta.hr	
HR metrics	NHR.0204.cv<br>NHR.0204.sd<br>NHR.0406.cv<br>NHR.0406.sd	
Sleep metrics	sleep.midpoint<br>sleep.offset.wd.sd	
Circadian rhythm – nonparametric	AC.hr.30m.wd<br>AC.hr.60m.wd<br>AC.st.30m<br>AC.st.60m	≥0.01 & <.05
Circadian rhythm – cosinor-based	acro.hr<br>acro.hr.wd<br>acro.st<br>beta.hr.wd<br>Pseudo-F.hr<br>Pseudo-F.hr.wd<br>Pseudo-F.st.wd	
HR metrics	DHR.cv<br>NHR.0002.cv<br>NHR.0002.sd	
Sleep metrics	sleep.midpoint.wd<br>sleep.midpoint.wd.sd<br>sleep.offset<br>sleep.offset.wd	

Table S5. Sociodemographic characteristics of participants from the contrasted subsample (depressed group has either baseline or follow up PHQ-9 score ≥10) in comparison to the whole set

Participants' characteristics	Contrasted subsample (n=78)	Full sample (n=267)	*P* value
Age (years), mean (SD)	31.6 (7.7)	32.8 (8.6)	.37 [a]
**Gender, n (%)**			
Male	29 (37%)	97 (36%)	.89 [b]
Female	49 (63%)	170 (64%)	
**Ethnicity, n (%)**			
Chinese	57 (73%)	211 (79%)	.53 [b]
Indian	9 (12%)	22 (8%)	
Malay	5 (6%)	10 (4%)	
Other	7 (9%)	24 (9%)	
**Marital status, n (%)**			
Married	30 (38%)	104 (39%)	1 [b]
Single	48 (62%)	163 (61%)	
**Education, n (%)**			
University degree	69 (88%)	238 (89%)	.84 [b]
Below university degree	9 (12%)	29 (11%)	
**Monthly income (SGD), n (%)**			
< 4,000	44 (56%)	154 (58%)	.90 [b]
≥ 4,000	34 (44%)	113 (42%)	

[a] Kruskal–Wallis rank sum test;
[b] Fisher's Exact Test.

Table S6. Sociodemographic characteristics of participants from the contrasted subsample (depressed group has both baseline and follow up PHQ-9 score ≥8) in comparison to the whole set

Participants' characteristics	Contrasted subsample (n=44)	Full sample (n=267)	*P* value
Age (years), mean (SD)	30.4 (7.8)	32.8 (8.6)	.044 [a]
**Gender, n (%)**			
Male	12 (27%)	97 (36%)	.31 [b]
Female	32 (73%)	170 (64%)	
**Ethnicity, n (%)**			
Chinese	31 (70%)	211 (79%)	.15 [b]
Indian	5 (11%)	22 (8%)	
Malay	5 (11%)	10 (4%)	
Other	3 (7%)	24 (9%)	
**Marital status, n (%)**			
Married	11 (25%)	104 (39%)	.09 [b]
Single	33 (75%)	163 (61%)	
**Education, n (%)**			
University degree	37 (84%)	238 (89%)	.32 [b]
Below university degree	7 (16%)	29 (11%)	
**Monthly income (SGD), n (%)**			
< 4,000	29 (66%)	154 (58%)	.33 [b]
≥ 4,000	15 (34%)	113 (42%)	

[a] Kruskal–Wallis rank sum test;

[b] Fisher's Exact Test.

Table S7. Sociodemographic characteristics of participants from the contrasted subsample (depressed group has the average PHQ-9 score ≥8) in comparison to the whole set

Participants' characteristics	Contrasted subsample (n=84)	Full sample (n=267)	*P* value
Age (years), mean (SD)	31.0 (7.8)	32.8 (8.6)	.09 [a]
**Gender, n (%)**			
Male	27 (32%)	97 (36%)	.52 [b]
Female	52 (68%)	170 (64%)	
**Ethnicity, n (%)**			
Chinese	64 (76%)	211 (79%)	.75 [b]
Indian	8 (10%)	22 (8%)	
Malay	5 (6%)	10 (4%)	
Other	7 (8%)	24 (9%)	
**Marital status, n (%)**			
Married	29 (35%)	104 (39%)	.52 [b]
Single	55 (65%)	163 (61%)	
**Education, n (%)**			
University degree	74 (88%)	238 (89%)	.84 [b]
Below university degree	10 (12%)	29 (11%)	
**Monthly income (SGD), n (%)**			
< 4,000	49 (58%)	154 (58%)	1 [b]
≥ 4,000	35 (42%)	113 (42%)	

[a] Kruskal–Wallis rank sum test;
[b] Fisher's Exact Test.

Table S8. Sociodemographic characteristics of participants from the contrasted subsample (top and bottom 20% by the average PHQ-9 score) in comparison to the whole set

Participants' characteristics	Contrasted subsample (n=96)	Full sample (n=267)	*P* value
Age (years), mean (SD)	31.7 (7.5)	32.8 (8.6)	.48 [a]
**Gender**, n (%)			
Male	48 (50%)	97 (36%)	.02 [b]
Female	48 (50%)	170 (64%)	
**Ethnicity**, n (%)			
Chinese	72 (75%)	211 (79%)	.58 [b]
Indian	10 (10%)	22 (8%)	
Malay	2 (2%)	10 (4%)	
Other	12 (13%)	24 (9%)	
**Marital status**, n (%)			
Married	34 (35%)	104 (39%)	.62 [b]
Single	62 (65%)	163 (61%)	
**Education**, n (%)			
University degree	86 (90%)	238 (11%)	1 [b]
Below university degree	10 (10%)	29 (89%)	
**Monthly income (SGD)**, n (%)			
< 4,000	50 (52%)	154 (58%)	.40 [b]
≥ 4,000	46 (48%)	113 (42%)	

[a] Kruskal–Wallis rank sum test;
[b] Fisher's Exact Test.

Table S9. Performance of depression screening status classification in the contrasted subsample with different feature subsets varying by categories

Model (feature set)	No info ratio	Acc	Sens	Spec	PPV	NPV	Kappa	AUC
All included	0.5	0.663	0.673	0.653	0.66	0.667	0.327	0.69
Circadian rhythm: nonparametric	0.5	0.684	0.612	0.755	0.714	0.661	0.367	0.68
Circadian rhythm: cosinor	0.5	0.663	0.592	0.735	0.69	0.643	0.327	0.65
HR metrics	0.5	0.704	0.714	0.694	0.7	0.708	0.408	0.72
Sleep metrics	0.5	0.612	0.612	0.612	0.612	0.612	0.224	0.6
Activity metrics	0.5	0.622	0.694	0.551	0.607	0.643	0.245	0.61
