# Supplementary material for: Digital Biomarkers for Depression Screening With Wearable Devices: Cross-sectional Study With Machine Learning Modeling
Source: JMIR Mhealth Uhealth. 2021 Oct 25;9(10):e24872. doi: 10.2196/24872 (PMC8576601; doi:10.2196/24872)
Supplement: Multimedia Appendix 2 [file mhealth_v9i10e24872_app2.pdf]

Multimedia Appendix 2. Digital biomarkers

Table S1. List of digital biomarkers with definitions

Category	Metric	Definition and interpretation	Data types and samples	Digital biomarkers (short names)
Physical activity metrics	Daily steps	Average of daily sum of steps over the observation period.	Total, and sampled from weekdays and weekends separately	steps<br>steps.wd<br>steps.we
	Sedentary time	Average daily sedentary time over the observation period. Sedentary time defined as waking behaviour characterized by an energy expenditure ≤1.5 metabolic equivalents (METs) [1].	Total, and sampled from weekdays and weekends separately	Sedentary<br>Sedentary.wd<br>Sedentary.we
	Light-intensity physical activity (LPA) - mean	Average daily time of physical activity of intensity below 3 METs over the observation period (according to the CDC's physical activity guidelines) [2]	Total	LPA
	Moderate-intensity physical activity (MPA) - mean	Average daily time of physical activity of intensity from 3 to 6 METs over the observation period (according to the CDC's physical activity guidelines) [2]	Total	MPA
	Vigorous-intensity physical activity (VPA) - mean	Average daily time of physical activity of intensity above 6 METs over the observation period (according to the CDC's physical activity guidelines) [2]	Total	VPA
Heart rate metrics	HR - mean, SD, and CV	Average, SD, and CV of HR (measured in beats per minute) over the observation period. SD and CV indicate the extent of stability/variability in HR.	Total	HR<br>HR.sd<br>HR.cv
	Resting HR - mean, SD, and CV	Average, SD, and CV of HR sampled from 15-minute intervals with zero steps over the observation period.	Total	RHR<br>RHR.sd<br>RHR.cv
	Delta of resting HR	The difference between average HR and resting HR	Total	dRHR
	Daytime HR - mean, SD, and CV	HR at day-time interval; mean, SD, and CV over the observation period were taken (determined according to [3])	HR data sampled from 2:00−4:00 pm	DHR<br>DHR.sd<br>DHR.cv
	Nighttime HR - mean, SD, and CV	HR at night-time intervals; mean, SD, and CV over the observation period were taken.	HR data sampled from three consecutive two-hour nighttime windows: 00:00−2:00 am, 2:00−4:00 am, and 4:00−6:00 am	NHR.0002<br>NHR.0002.sd<br>NHR.0002.cv<br>NHR.0204<br>NHR.0204.sd<br>NHR.0204.cv<br>NHR.0406<br>NHR.0406.sd<br>NHR.0406.cv

	Root mean square of successive differences of HR	The root mean square of successive differences of HR over the observation period; the metric characterizes the sharpness of successive HR deviations and can be interpreted as a proxy measure of ECG-based HR variability.	Raw HR data and HR averaged by hourly	HR.rmssd<br>HR.rmssd.h
COSINOR rhythm metrics *	Acrophase	The time of day of the cosine curve peak (measured in hours since midnight).	Steps data (hourly intervals), HR data (hourly intervals), total, weekdays	acrophase.st<br>acrophase.st.wd<br>acrophase.hr<br>acrophase.hr.wd
	MESOR	The Midline Estimating Statistic of Rhythm, a rhythm-adjusted mean value which estimates central tendency of the distribution of an oscillating variable [4].	Steps data (hourly intervals), HR data (hourly intervals), total, weekdays	mesor.st<br>mesor.st.wd<br>mesor.hr<br>mesor.hr.wd
	Amplitude	The difference between the maximum value of the fitted cosine curve and MESOR. The lower amplitude indicates a more dampened rhythm.	Steps data (hourly intervals), HR data (hourly intervals), total, weekdays	amp.st<br>amp.st.wd<br>amp.hr<br>amp.hr.wd
	Pseudo F-statistic	Pseudo F-statistic of a model's goodness of fit, which indicate, so-called, robustness of the rhythm.	Steps data (hourly intervals), HR data (hourly intervals), total, weekdays	F.st<br>F.st.wd<br>F.hr<br>F.hr.wd
	α		Steps data (hourly intervals), HR data (hourly intervals), total, weekdays	alpha.st<br>alpha.st.wd<br>alpha.hr<br>alpha.hr.wd
	β		Steps data (hourly intervals), HR data (hourly intervals), total, weekdays	beta.st<br>beta.st.wd<br>beta.hr<br>beta.hr.wd
Nonparametric rhythm metrics	Inter-daily Stability (IS)	The ratio of variance of the average 24h data profile to the total variance of all days (data are summarized by hourly)<br>$$\mathrm{IS} = \frac{\sum_{h=1}^{p} (x_h - \bar{x})^2 N}{\sum_{i=1}^{N} (x_i - \bar{x})^2 p}$$<br>in which N is the total number of data items, p is the number of data items per day (24 in this case), x_h corresponds to each hour of the mean profile, while x_i represents each given hour of raw data, and $\bar{x}$ is the average of all data [5].<br>IS is a measure of stability/regularity of circadian rhythm over a series of 24h cycles, i.e. the extent to which a day-by-day activity follows some regular pattern, where higher values indicate a more stable circadian	Steps data (hourly intervals), HR data (hourly intervals), total, weekdays	IS.st<br>IS.st.wd<br>IS.hr<br>IS.hr.wd

		rhythm.		
	Intra-daily Variability (IV)	The mean square of differences between successive hourly data (i.e., the first derivative) normalized by the total variance of all days (data are summarized by hourly) $$\mathrm{IV} = \frac{\sum_{i=2}^{N} (x_i - x_{i-1})^2\, N}{\sum_{i=1}^{N} (x_i - \bar{x})^2\, (N-1)}$$ in which N is the total number of data items, x_i represents data point of a given hour, and $\bar{x}$ is the average of all data. IV quantifies the fragmentation of periods of activity from periods of rest within a 24-h cycle [5]. Intra-daily variability scores range from 0 to 2 and are typically below 1, where higher values indicate a more fragmented rhythm and reflect shorter alternating periods of rest and activity rather than one extended active period during the daytime and one extended rest period at night.	Steps data (hourly intervals), HR data (hourly intervals), total, weekdays	IV.st<br>IV.st.wd<br>IV.hr<br>IV.hr.wd
	M10	Diurnal activity, the mean activity of the ten consecutive most active hours of the average daily activity profile.	Steps data (hourly intervals), HR data (by-minute intervals), total, weekdays	M10.st<br>M10.st.wd<br>M10.hr<br>M10.hr.wd
	L5	Nocturnal activity, the mean activity of the five consecutive least active hours of the average daily activity profile.	Steps data (hourly intervals), HR data (by-minute intervals), total, weekdays	L5.st<br>L5.st.wd<br>L5.hr<br>L5.hr.wd
	Relative amplitude	The difference between M10 and L5 divided by the sum of M10 and L5, theoretically range from 0 to 1, where higher values results from greater daytime activity and reduced activity during sleep and shows greater amplitude. $$RA = \frac{M10 - L5}{M10 + L5}$$	Steps data, HR data, total, weekdays	RA.st<br>RA.st.wd<br>RA.hr<br>RA.hr.wd
	Inter-daily coefficient of variation (ICV)	The 24h mean of by-hour coefficients of variation (CV), where CV is the ratio of SD to average in each hour between days. $$\mathrm{ICV} = \frac{1}{p}\sum_{h=1}^{p} \frac{\sqrt{\frac{\sum_{i=1}^{N} (x_i - x_h)^2}{N}}}{x_h}$$ in which p is the number of data points per day (24 in this case, data is	Steps data, HR data, total, weekdays	ICV.st<br>ICV.st.wd<br>ICV.hr<br>ICV.hr.wd

		aggregated by-hourly), x_i represents values corresponding to each hour from all days, x_h represents values of each hour from the mean 24h profile, and *N* is the number of days. Higher ICV indicates higher variation and less stable rhythm. We proposed this metric as alternative to IS, which assesses rhythm stability with a different approach.		
	Rhythm autocorrelation (AC)	The autocorrelation of time series with a day-length lag, another alternative measure of a rhythm stability; higher values indicate higher stability/similarity of data patterns across days. $$\mathrm{AC} = \frac{\sum_{i=1}^{N-k}(x_i - \bar{x})(x_{i+k} - \bar{x})}{\sum_{i=1}^{N}(x_i - \bar{x})^2}$$ in which k is a day-length lag (e.g. 24 if data is aggregated by-hourly), x_i represents values of each interval, $\bar{x}$ the average of all data, and *N* is total number of data points.	Steps data (aggregated into hourly intervals, 30-minute intervals, and 15-minute intervals), HR data (aggregated into hourly intervals, 30-minute intervals, and 15-minute intervals), total, weekdays	AC.st.60m<br>AC.st.30m<br>AC.st.15m<br>AC.st.60m.wd<br>AC.st.30m.wd<br>AC.st.15m.wd<br>AC.hr.60m<br>AC.hr.30m<br>AC.hr.15m<br>AC.hr.60m.wd<br>AC.hr.30m.wd<br>AC.hr.15m.wd
Peak detection-based metrics	Daily peaks - mean, and SD	The number of peaks per day in time series. Robust peak detection algorithm was used to identify peaks (following algorithm parameters were set for steps data: lag = 10, threshold = 10, influence = 0; for HR data: lag = 10, threshold = 2, influence =0.25) [6]; average and SD over the observation period were taken.<br>Peaks indicate and quantify distinct behavioral and physiological activities happening over a day or transitions between these activities, while SD and CV are stability estimates.	Steps data (aggregated into 15-minute intervals, intervals with less than 50 steps were recoded as zeros to reduce noise), HR data (aggregated into 15-minute intervals), total, weekdays	peaks.st<br>peaks.st.sd<br>peaks.st.wd<br>peaks.st.wd.sd<br>peaks.hr<br>peaks.hr.sd<br>peaks.hr.wd<br>peaks.hr.wd.sd
Sleep metrics **	Time in bed (TB) - mean and CV	The length of time in bed over the observation period; average and CV over the observation period. Measured in minutes.	All data, weekdays	TB<br>TB.cv<br>TB.wd<br>TB.wd.cv
	Total sleep time (TST) - mean and CV	The differences between the length of time in bed and length of wake time; average and CV over the observation period. Measured in minutes.	All data, weekdays	TST<br>TST.cv<br>TST.wd<br>TST.wd.cv
	Sleep efficiency (SE) - mean and CV	The ratio of total sleep time to time in bed; average and CV over the observation period.	All data, weekdays	SE<br>SE.cv<br>SE.wd<br>SE.wd.cv
	Sleep onset latency (SOL) - mean and CV	The length of time until the first minute of a sleep onset; average over the observation period. Measured in minutes.	All data, weekdays, light sleep	SOL<br>SOL.cv

				SOL.wd<br>SOL.wd.cv
	Wake after sleep onset (WASO) - mean and CV	Sum of wake minutes in the middle half of sleep episode (within second and third quartiles of a sleep episode).	All data, weekdays	WASO<br>WASO.cv<br>WASO.wd<br>WASO.wd.cv
	Sleep offset - mean and SD	Awakening time after a sleep; average over the observation period. Sleep offset time is measured in hours since midnight, while SD is in minutes.	All data, weekdays	sleep.offset<br>sleep.offset.sd<br>sleep.offset.wd<br>sleep.offset.wd.sd
	Sleep midpoint - mean and SD	Time of a sleep midpoint; average over the observation period. Sleep midpoint is measured in hours since midnight, while SD is in minutes.	All data, weekdays	sleep.midpoint<br>sleep.midpoint.sd<br>sleep.midpoint.wd<br>sleep.midpoint.wd.sd

* Cosine curve was fitted using data aggregated by-hourly.

** Sleep metrics derived from sleep stages which are determined algorithmically by manufacturer based on sensor data. Fitbit wearables identify each 30-second interval of sleep episode as light sleep, deep sleep, Rapid eye movement (REM) sleep or wake.

Abbreviations: HR- heart rate; SD – standard deviation; CV – coefficient of variation (the ratio of a SD to a mean); REM – Rapid Eye Movement; SOL - sleep onset latency; TB – time in bed; TST – total sleep time; IS – inter-daily stability; IC – intra-daily variation; ICV – inter-daily coefficient of variation; SE – sleep efficiency; LPA – light-intensity physical activity; MPA – moderate-intensity physical activity; VPA – vigorous-intensity physical activity.

References

1. Tremblay MS, Aubert S, Barnes JD, Saunders TJ, Carson V, Latimer-Cheung AE, et al. Sedentary Behavior Research Network (SBRN) – Terminology Consensus Project process and outcome. International Journal of Behavioral Nutrition and Physical Activity 2017 Jun 10;14(1):75. [doi: 10.1186/s12966-017-0525-8]

2. U.S. Department of Health and Human Services, Public Health Service, Centers for Disease Control and Prevention, National Center for Chronic Disease Prevention and Health Promotion, Division of Nutrition and Physical Activity. Promoting Physical Activity: A Guide for Community Action. Champaign, IL: Human Kinetics; 1999.

3. Lim WK, Davila S, Teo JX, Yang C, Pua CJ, Blöcker C, et al. Beyond fitness tracking: The use of consumer-grade wearable data from normal volunteers in cardiovascular and lipidomics research. PLOS Biology 2018 Feb 27;16(2):e2004285. [doi: 10.1371/journal.pbio.2004285]

4. Cornelissen G. Cosinor-based rhythmometry. Theoretical Biology and Medical Modelling 2014 Apr 11;11(1):16. [doi: 10.1186/1742-4682-11-16]

5. Gonçalves B, Adamowicz T, Louzada FM, Moreno CR, Araujo JF. A fresh look at the use of nonparametric analysis in actimetry. Sleep Medicine Reviews 2015 Apr 1;20:84–91. [doi: 10.1016/j.smrv.2014.06.002]

6. Brakel J-P. Peak signal detection in realtime timeseries data: robust peak detection algorithm (using z-scores) [Internet]. Stack Overflow. [cited 2020 Jul 2]. Available from: https://stackoverflow.com/questions/22583391/peak-signal-detection-in-realtime-timeseries-data
