# Supplementary figures and images for: Digital Biomarkers for Depression Screening With Wearable Devices: Cross-sectional Study With Machine Learning Modeling
Source: JMIR Mhealth Uhealth. 2021 Oct 25;9(10):e24872. doi: 10.2196/24872 (PMC8576601; doi:10.2196/24872)

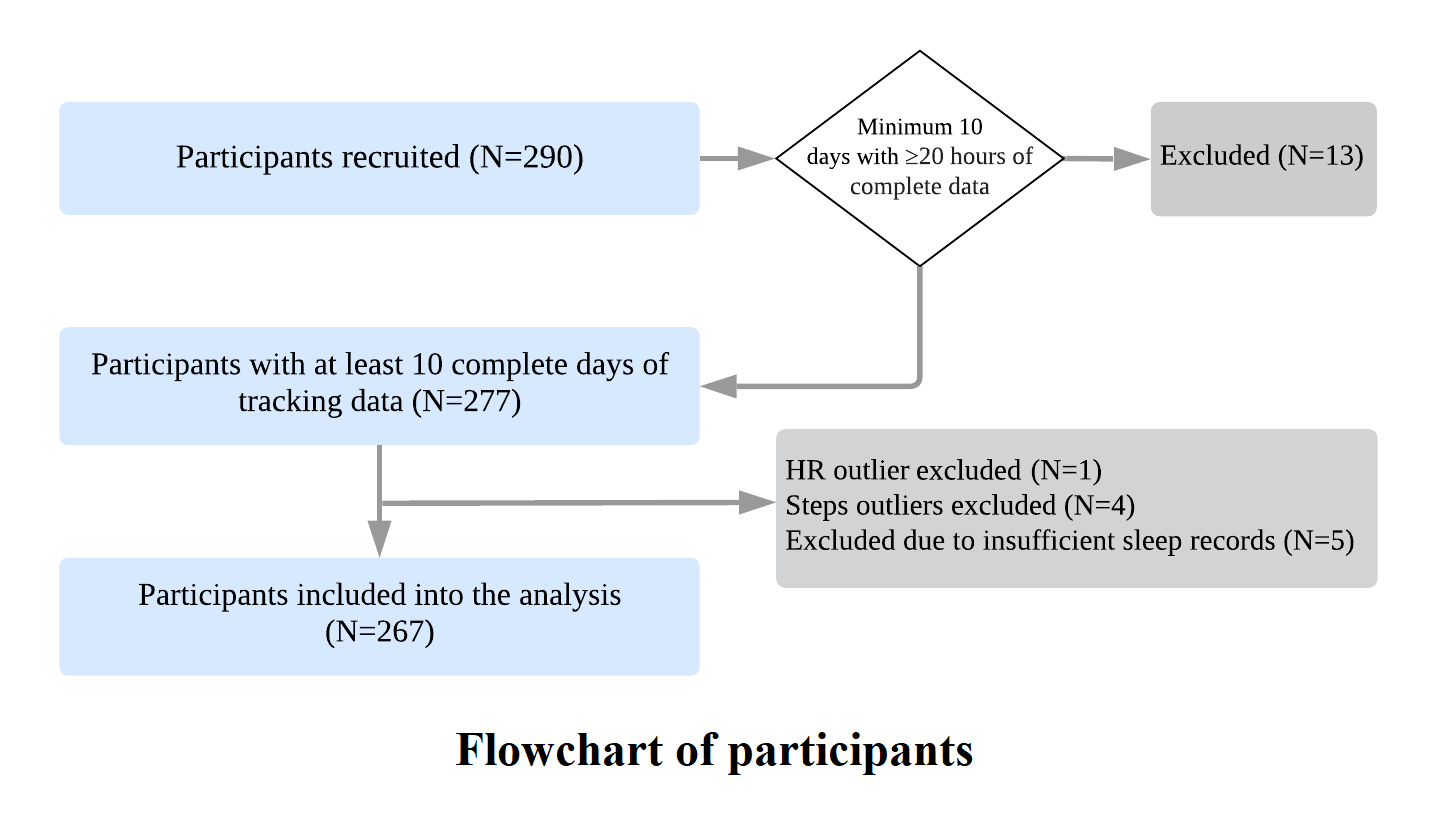

Supplement: Multimedia Appendix 4 [file mhealth_v9i10e24872_app4.png]
